# Deconstructing cancer in 3D: models, mechanisms, and personalized solutions

**DOI:** 10.1186/s12943-026-02704-8

**Published:** 2026-06-05

**Authors:** Zhao Huang, Xirui Duan, Jun Ji, Qu Cai, Ping Jin, Beiqin Yu, Jun Zhang

**Affiliations:** 1 West China Institute of Preventive and Medical Integration for Major Diseases, West China School of Public Health and West China Fourth Hospital, Sichuan University, Chengdu, Sichuan China; 2 Department of Oncology, Ruijin Hospital, Shanghai Jiao Tong University School of Medicine, Shanghai, China; 3 Shanghai Institute of Digestive Surgery, Ruijin Hospital, Shanghai Jiao Tong University School of Medicine, Shanghai, China; 4 State Key Laboratory for Conservation and Utilization of Bio-Resources in Yunnan and Key Laboratory of Industrial Microbial Fermentation Engineering of Yunnan Province, School of Life Sciences, Yunnan University, Kunming, China; 5 Department of General Surgery, Ruijin Hospital, Shanghai Jiao Tong University School of Medicine, Shanghai, China; 6 Shanghai Key Laboratory of Gastric Neoplasms, Ruijin Hospital, Shanghai Jiao Tong University School of Medicine, Shanghai, China

**Keywords:** 3D cancer models, Patient-derived organoids, Tumor microenvironment, Precision medicine, Drug resistance

## Abstract

Three-dimensional (3D) cancer models, notably patient-derived organoids (PDOs), address the critical limitations of traditional preclinical systems, including two-dimensional (2D) monolayer cultures and patient-derived xenografts (PDXs), by better recapitulating physiological tumor architecture and patient-specific heterogeneity, thereby revolutionizing oncology research. We chart the complementary technological landscape, from high-fidelity PDOs and scalable spheroids to engineered systems that reconstruct the tumor microenvironment (TME) via co-culture, organ-on-a-chip, and 3D bioprinting. Beyond foundational biology, these tools are driving functional precision medicine, where PDO avatars predict clinical drug response, and accelerating drug discovery through physiologically relevant screening. A core focus is their unparalleled utility in modeling therapy resistance, enabling the induction and multi-omic dissection of resistant clones, deconstructing stroma-mediated protection, and testing rational combination therapies to overcome relapse. Despite challenges in standardization and complete TME integration, the convergence of 3D models with single-cell omics, CRISPR screening, and artificial intelligence heralds a new era of predictive oncology. Ultimately, rigorous validation and clinical translation of these models promise to bridge the gap between bench and bedside, enabling truly personalized and effective cancer therapies.

## Introduction

The relentless pursuit of effective cancer therapies is fundamentally constrained by the fidelity of the models used to interrogate tumor biology and predict clinical outcomes. For decades, the preclinical oncology research paradigm has rested upon two primary pillars: two-dimensional (2D) monolayer cultures of immortalized cancer cell lines and patient-derived xenografts (PDXs) established in immunocompromised mice. While these systems have yielded foundational insights and catalyzed drug discovery, their profound and inherent limitations are now starkly apparent, directly contributing to the staggeringly high failure rates of oncology drug candidates in clinical trials. Specifically, 2D monolayers lack physiological tissue architecture, nutrient and oxygen gradients, and cell‑polarity signaling, leading to false‑positive drug activity that does not translate into solid tumors. For instance, lung adenocarcinoma cells grown in 2D become artificially dependent on EGFR signaling and show misleading sensitivity to EGFR inhibitors, whereas the same cells in three-dimensional (3D) spheroid culture retain microRNA‑mediated feedback regulation and are far less vulnerable to inhibition [1]. As to PDXs, they lack a functional human immune system and progressively replace human stroma with murine cells, rendering them incapable of modeling immunotherapies or human‑specific stromal contributions to resistance. Genome‑wide analyses show that 41–89% of transcription factor binding sites differ between humans and mice, leading to altered gene expression programs that govern drug metabolism, signaling adaptation, and tumor-stroma crosstalk [2]. The central challenge lies in their inability to capture the intricate, multidimensional reality of human cancer as a complex tissue ecosystem. The simplification imposed by conventional two-dimensional culture is drastic. On the rigid, homogeneous surface of a plastic dish, cancer cells adopt unnatural morphologies, lose critical cell-polarity signaling, and experience uniform exposure to nutrients and oxygen [3]. This aberrant environment precipitates widespread changes in gene expression, metabolic pathways, and proliferation dynamics that diverge significantly from in vivo physiology [4]. Consequently, drug sensitivity observed in 2D often bears little resemblance to therapeutic response in patients, as factors central to drug efficacy, such as penetration gradients, cell-ECM adhesion-mediated survival signals, and the spatial organization of proliferative versus quiescent cell populations, are entirely absent [5]. This discrepancy renders 2D models inadequate for reliable prediction of compound efficacy, toxicity, and the inevitable emergence of resistance. Systematic comparisons have shown that drug sensitivity profiles in 2D and 3D cultures differ markedly and unpredictably across cell lines and compounds, with no consistent scaling factor to correct for the discrepancy [4]. For example, A549 lung cancer cells are sensitive to the EGFR inhibitor erlotinib in 2D (IC50 about 5 µM) but completely resistant in 3D spheroid culture (IC50 > 50 µM), a difference driven by reduced AKT-mTOR-S6K signaling in the 3D context. Such profound and context-dependent divergence observed across multiple cell lines and drugs means that reliance on 2D assays alone will generate both false positives (compounds that appear active only in 2D) and false negatives (compounds that appear inactive in 2D but would be effective in vivo), undermining their predictive utility for clinical translation [4]. PDX models emerged as a superior alternative by implanting fragments of human tumor tissue into mice, typically under the renal capsule or in the mammary fat pad [6]. PDXs demonstrably retain the histopathological architecture, genomic landscape, and often the intratumoral heterogeneity of the original patient tumor, offering a more biologically relevant platform for studying tumor growth and some aspects of drug response [7]. However, the PDX system introduces its own set of formidable constraints. The engraftment process is slow, expensive, and suffers from variable take rates, making high-throughput studies impractical. Reported take rates vary widely by tumor type, ranging from 70 to 90% for colorectal cancer to only 20–30% for pancreatic cancer and 20–50% for breast cancer; the time from implantation to usable model typically spans 2–8 months, with associated costs substantially exceeding those of cell line-based platforms [8, 9]. The use of immunodeficient mouse hosts represents perhaps the most significant shortfall, as it completely eliminates the possibility of studying the dynamic interplay between human tumor cells and the adaptive and innate immune system, which is now recognized as paramount for the efficacy of modern immunotherapies and a key contributor to the tumor microenvironment [10]. Furthermore, the murine stromal cells that eventually replace the human tumor stroma create a chimeric microenvironment that may unpredictably influence tumor behavior and drug response [11].

These model deficiencies are particularly problematic in the face of our contemporary understanding of cancer biology. A tumor is not merely an aggregate of malignant cells but a complex, adaptive “organ” comprised of diverse cellular and acellular components collectively termed the tumor microenvironment [12]. This TME includes cancer-associated fibroblasts that remodel the extracellular matrix and secrete pro-survival signals, endothelial cells forming often dysfunctional vasculature, and a plethora of immune cells whose functional states can either antagonize or promote tumor progression. Critically, the TME is not a passive bystander but an active architect of therapy resistance. It can create physical barriers to drug delivery, provide sanctuary niches for drug-tolerant persister cells, and establish an immunosuppressive milieu that renders immunotherapies ineffective [13]. Therefore, a model that fails to incorporate these elements is, by design, incapable of unraveling the mechanisms of treatment failure or accurately predicting the success of novel strategies aimed at reprogramming the TME. This pressing need has catalyzed a paradigm shift toward the development and refinement of sophisticated three-dimensional in vitro cancer models. The 3D model landscape is rich and varied, encompassing everything from self-aggregating multicellular spheroids to patient-derived organoids (PDOs) that exhibit remarkable self-organization, and extending to bioengineered constructs that use microfabrication and bioprinting to impose precise spatial control over multiple cell types and matrix properties [14]. These models collectively offer unprecedented opportunities to deconstruct the multicellular complexity of tumors, control experimental variables with precision, and perform scalable, high-content functional analyses.

This review provided a comprehensive analysis of this transformative technological frontier. The technical principles, strengths, and current limitations of the major classes of 3D cancer models were introduced, with a particular focus on PDOs as a central pillar. Then, their transformative applications in oncology, especially their role in functional precision medicine where they accelerate the drug discovery pipeline, were discussed. A major highlight was dedicated to exploring how these 3D systems are uniquely positioned to illuminate the dark corners of therapy resistance, enabling the study of adaptive responses within a structured TME context. Finally, we also summarized the ongoing challenges in standardization, scalability, and complete TME recapitulation, and project a future where the integration of 3D models with cutting-edge technologies like single-cell multi-omics, CRISPR-based screening, and artificial intelligence will usher in a new era of predictive and personalized cancer research.

## The technological landscape of 3D cancer models

**Fig. 1:**
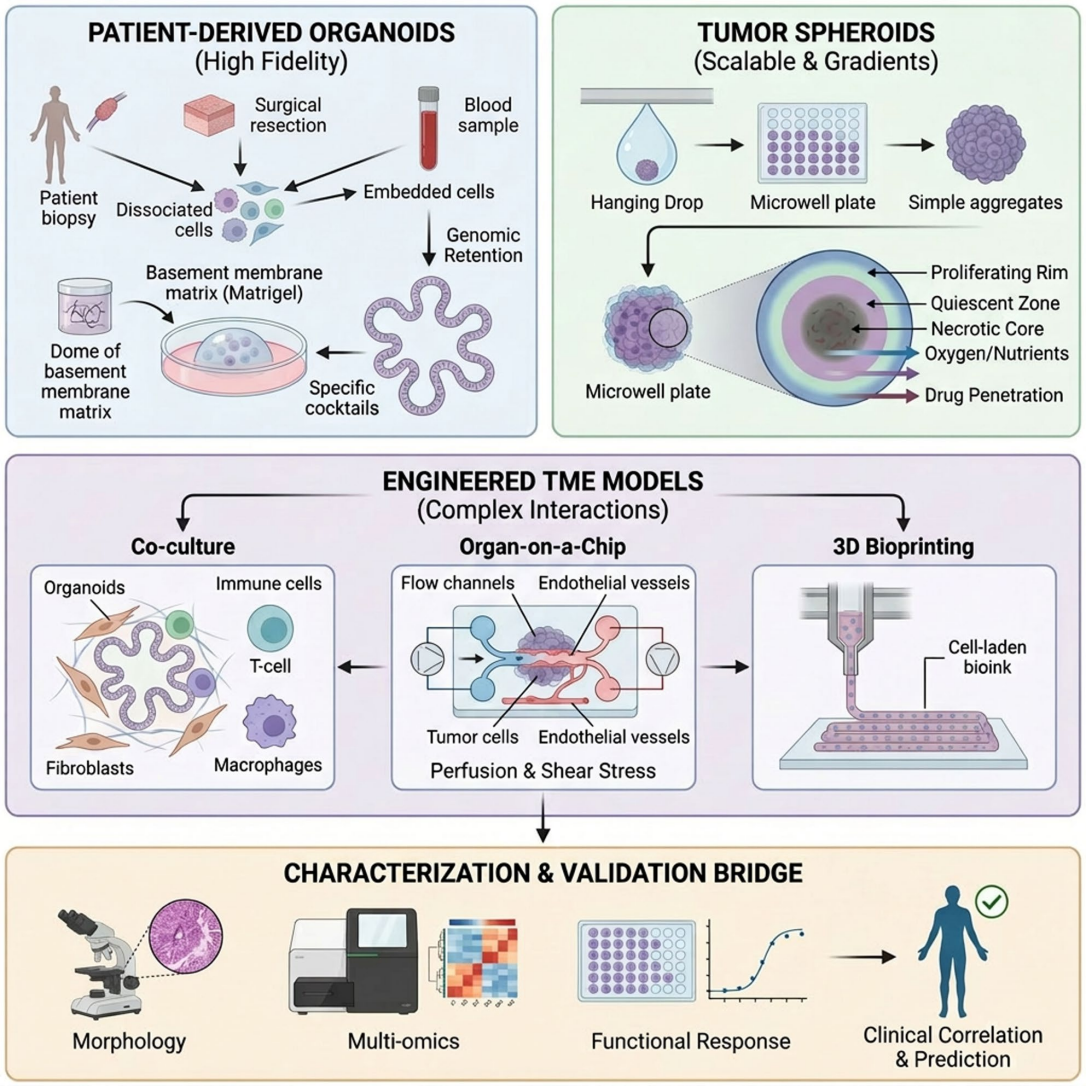
Technological landscape and validation framework of 3D cancer models. Conceptual overview of complementary three-dimensional (3D) in vitro cancer platforms spanning increasing degrees of biological fidelity, scalability and microenvironmental control. PDOs are established from clinical specimens and preserve key genomic, molecular and histopathological features of the source tumor, enabling patient-specific modeling. Tumor spheroids, generated by scaffold-free aggregation, recapitulate size-dependent proliferative, quiescent and necrotic gradients and provide scalable systems for mechanistic studies and drug screening. Engineered tumor microenvironment models including stromal and immune co-cultures, microfluidic organ-on-a-chip systems and spatially defined 3D bioprinting introduce controlled multicellular interactions, perfusion and architectural precision. Integrated morphological, multi-omics and functional characterization links these platforms to clinical correlation and predictive applications, forming a translational bridge between in vitro modeling and precision oncology

The quest to model cancer in vitro has evolved from simple cellular monolayers to a sophisticated array of three-dimensional systems, each designed to capture specific facets of tumor biology with varying degrees of complexity and control. This technological landscape is not a linear hierarchy but a complementary toolkit, where the choice of model is dictated by the specific research question, namely whether it demands high-throughput screening, unparalleled patient-specific fidelity, or the precise engineering of multicellular interactions. The following sections provide a critical overview of these core platforms, beginning with the gold standard of biological authenticity, moving through the versatile and accessible workhorse, and culminating in the frontier of engineered systems that seek to reconstruct the dynamic tumor microenvironment. Finally, we address the crucial framework for validating these complex models to ensure their outputs are meaningful and reproducible (Fig. 1).

### Patient-derived organoids: a patient-specific avatar model

Patient-derived organoids (PDOs) represent the most significant breakthrough in recapitulating human cancer biology in vitro [15]. Unlike cell lines, which are clonal populations adapted to plastic, PDOs are three-dimensional, self-organizing structures derived directly from patient tissue samples including surgical resections, biopsies, or even circulating tumor cells. Their fundamental premise is to leverage the intrinsic growth and self-renewal capacities of cancer cells within a controlled, three-dimensional extracellular matrix microenvironment that mimics key aspects of the native tissue niche.

The foundational protocol for establishing epithelial organoids involves embedding dissociated tissue fragments or single cells within a dome of basement membrane extract, most commonly Matrigel [16]. This matrix provides crucial biochemical cues such as laminin and collagen IV and physical scaffolding [17]. Though Matrigel has long been proved effective in PDO establishment, however, Matrigel contains thousands of exogenous proteins which introduce large variations and difficulties for elucidating physiological cues necessary for the development and function of PDOs [18]. To better mimic physiological context, organoid culture without Matrigel has been recently developed [19]. For example, Matrigel-free prostate cancer (PCa) PDOs exhibit active androgen receptor signaling which reflects patient-specific characteristics, whereas PCa PDOs developed in conventional Matrigel produce basal-like features which is distinct with primary PCa cells derived from patients [20]. Therefore, a Matrigel-free system might be more suitable for the construction of native pathophysiological environment in PDO culture.

Beyond the choice of matrix as a passive scaffold, the extracellular matrix (ECM) itself actively shapes tumor phenotype, signaling, and drug response, and should therefore be considered a model‑defining determinant of translational fidelity [21]. ECM provenance matters: animal‑derived matrices such as Matrigel contain thousands of exogenous proteins and exhibit batch‑to‑batch variability, complicating mechanistic interpretation and reproducibility. By contrast, synthetic hydrogels offer defined biochemical and mechanical properties, enabling systematic decoupling of individual ECM cues [22]. ECM mechanics are equally critical; matrix stiffness, viscoelasticity, and degradability influence cancer cell proliferation, invasion, and therapeutic sensitivity through mechanotransduction pathways such as YAP/TAZ signaling [23]. Using dynamically crosslinked hydrogels, recent studies have demonstrated that viscoelastic properties, particularly stress relaxation, modulate invasive behavior, stem‑like marker expression, and drug responses in three‑dimensional cancer models [24]. In pancreatic cancer, type I collagen-rich dense stroma has been shown to limit drug penetration and promote metabolic adaptations that support chemoresistance [25]. Emerging strategies employ dense or spatially patterned matrices to model desmoplastic stroma, as well as engineered matrices with tunable ligand presentation to probe receptor‑mediated signaling. Recognizing ECM as an active, tunable determinant of cancer phenotype rather than an inert support is essential for improving model fidelity, inter‑laboratory reproducibility, and clinical translation.

Then, the samples are overlaid with a meticulously formulated culture medium which typically includes Wnt agonists like R-spondin-1, Noggin (a BMP inhibitor), and epidermal growth factor, alongside niche-specific factors such as FGF-10 for lung or hepatocyte growth factor for liver organoids. This organoid culture approach has now been successfully adapted to a vast array of solid tumors, including colorectal, pancreatic, prostate, breast, brain, liver, lung, gastric, and many other cancers [26–33]. However, the protocol is far from uniform, and success hinges on tumor-type-specific optimization. For instance, pancreatic ductal adenocarcinoma organoids often require the addition of TGF-β pathway inhibitors, while hormone-sensitive cancers like prostate or breast may need androgen or estrogen supplementation [34–36]. The source material also dictates methodology. Core needle biopsies, which yield limited tissue, require efficient single-cell dissociation and embedding protocols, whereas surgical specimens allow for both mechanical and enzymatic processing to generate multiple organoid lines from different regions of a single tumor, enabling the study of intratumoral heterogeneity. A critical and evolving subfield is the derivation of organoids from liquid biopsies, such as circulating tumor cells, which offers a minimally invasive, serial sampling opportunity to track clonal evolution and therapy response over time [37]. For example, PDOs derived from circulating breast cancer cells enable the identification of NRG1-HER3 axis as the key signaling driving breast cancer metastasis [38]. While technically demanding and currently characterized by lower success rates, this approach holds immense promise for monitoring metastatic disease and acquired resistance.

The paramount value of PDOs lies in their remarkable ability to preserve the defining molecular and histopathological features of the source tumor across multiple passages. Whole-exome and whole-genome sequencing analyses have consistently shown that PDOs retain the key driver mutations, copy number alterations, and structural variants of the parental tumor, including clinically actionable alterations [39, 40]. This mutational landscape remains stable over several months of expansion, although selective pressures in culture can, over very long periods, lead to the outgrowth of subclones [41]. RNA sequencing reveals that PDOs largely maintain the global gene expression signature and subtype-specific classifiers of their tissue of origin, preserving pathway activation states crucial for therapy response [42, 43]. Perhaps the most visually compelling evidence is the histological resemblance; hematoxylin and eosin staining of PDO sections often reveals recapitulation of tumor-specific architecture, and immunohistochemistry for lineage markers and key oncoproteins typically mirrors the original patient sample [44–46]. The ultimate validation is functional. A growing body of evidence demonstrates a significant correlation between drug sensitivity in PDOs and the clinical response observed in the corresponding patients received standard chemotherapies, targeted agents, and novel combinations with or without primary resistance or acquired resistance, and this issue will be discussed in later section.

### Tumor spheroids: a more accessible tool

While PDOs represent a pinnacle of biological fidelity, tumor spheroids constitute a more accessible, versatile, and historically established cornerstone of 3D cancer modeling [47]. Spheroids are defined as self-assembled, spherical aggregates of cancer cells that form under conditions preventing cellular adhesion to a rigid substrate [48]. They serve as a critical intermediate model, capturing essential 3D characteristics absent in monolayer culture, such as cell-cell adhesion, nutrient/oxygen diffusion gradients, and emergent collective behaviors, without the technical complexity and cost associated with organoid culture. Spheroids can be generated from established cancer cell lines, primary cell isolates, or co-cultures. The initial cell number dictates the final spheroid size, which in turn governs its internal architecture. Small spheroids are largely proliferative, while larger spheroids develop a characteristic layered structure including a proliferative outer rim, a quiescent middle region, and a necrotic core due to oxygen and nutrient diffusion limits [49]. This spontaneously arising physiological gradient mimics the micro-regions found in avascular tumor nodules or the interior of poorly perfused solid tumors, creating microenvironments of differential proliferation, metabolism, and drug sensitivity.

The formation of tumor spheroids exploits the innate tendency of many cell types to re-aggregate, and the choice of method balances throughput, size uniformity, and experimental requirements. The most common and straightforward technique is the liquid overlay method, where cells are seeded onto plates coated with non-adhesive hydrogels like agarose or into commercially available ultra-low attachment plates, clustering into spheroids over 24–72 h [50]. While simple, this method can yield heterogeneous spheroid sizes, and a modular framework can be adopted to analyze multifaceted phenotype of spheroids with high quality and reproducibility [51]. For superior size uniformity, the hanging drop method is employed, where a droplet of cell suspension forms a single, uniform spheroid per drop, though it is low-throughput and manually intensive [52]. In addition, when handling Petri dishes, there is a risk of droplet coalescence, making it difficult to generate numerous spheroids in a limited area. To address this issue, 3D printing-based support was created to avoid droplet coalescence thus improving the performance of this method [53]. Agitation-based methods using spinner flasks or orbital shakers allow for the generation of large quantities of spheroids and can scale up, but controlling size precisely is challenging [54]. As an alternative to these approaches, microcavity arrays offer a scalable and highly uniform platform for spheroid and organoid generation. These microfabricated devices contain thousands of microwells of defined geometry that physically confine cell aggregation, yielding spheroids with consistent size and shape while enabling automated medium exchange and high-content imaging. This technology bridges the gap between low-throughput hanging drop methods and poorly controlled agitation-based systems, and has been successfully applied to both cancer cell line-derived spheroids and PDOs [55].

The utility of tumor spheroids spans fundamental biology and applied pharmacology. They are a premier model for studying tumor microenvironment gradients, where the hypoxic core activates HIF-1α signaling, driving changes that promote chemoresistance [56]. Researchers can spatially map metabolites, pH, and proliferation markers within a single spheroid. The compact structure also makes them ideal for drug penetration and efficacy studies, simulating the delivery challenges faced in vivo. By treating spheroids with fluorescently tagged drugs and using confocal microscopy, researchers can quantify penetration kinetics and identify physical barriers, which is crucial for optimizing drug formulations [57]. A major application lies in high-throughput therapeutic screening. Their compatibility with automated liquid handling and high-content imaging systems enables medium- to high-throughput campaigns where viability assays can be performed in situ, allowing for the rapid assessment of hundreds of compounds on 3D structures in a format that is far more scalable than PDOs [58]. Furthermore, when embedded into a 3D matrix like collagen, invasive cancer cell spheroids serve as a controlled “micro-tumor” in the spheroid invasion assay, a workhorse for quantifying radial invasion and studying the molecular mechanisms underlying metastasis [59]. By analyzing fluorescent images at the pixel level, 3D spheroid cell invasion can be quantified in an automated, high-throughput manner, providing objective and consistent invasion metrics that minimize sensitivity to spheroid size and cell spreading [60].

The choice between spheroids and organoids is not a matter of superiority but of aligning the model with the scientific question, and a direct comparison clarifies their distinct niches. Biologically, spheroids are typically generated from established, homogenous cell lines, leading to aggregates that lack the genetic diversity and tissue-specific architecture of the original tumor. In contrast, organoids are derived directly from patient tissue and recapitulate glandular or other histopathological structures, possessing higher architectural and genetic fidelity. Regarding the tumor microenvironment, both models lack a full, physiological TME in their basic form, though the spheroid’s necrotic core effectively models metabolic stress gradients. Organoids are primarily epithelial structures where the TME must be intentionally added back via co-culture, whereas in spheroids, co-culture is straightforward by mixing cells before aggregation. In terms of throughput, cost, and accessibility, spheroids are the clear winner, requiring no specialized media and being compatible with high-throughput workflows, while organoid culture demands expertise and expensive reagents, limiting scalability. Consequently, spheroids excel as a generalizable 3D model system for studying universal cancer biology principles like gradients and drug penetration, and for scalable, first-pass 3D drug screening. Organoids excel as a patient-specific avatar model for personalized medicine, studying tumor-subtype-specific biology, and investigating driver mutation functionality. The most powerful research strategies often employ both in tandem, using spheroids for controlled, high-throughput mechanistic studies and organoids for translational validation and personalized prediction. Importantly, recent technological advances are beginning to address the scalability limitations of organoid culture, narrowing the throughput gap between spheroids and organoids. Suspension culture methods, in which organoids are grown in agitated bioreactors rather than embedded in stationary matrix domes, enable large-scale expansion and facilitate high-throughput drug perturbation screens [61]. Similarly, optimized media formulations and culture protocols have been developed that support long-term maintenance of patient-specific molecular and phenotypic characteristics across multiple cancer indications, improving the reproducibility and scalability of organoid-based platforms [62]. When combined with microcavity array technologies that enable automated organoid formation and processing, these approaches collectively enhance the feasibility of incorporating PDOs into medium- to high-throughput screening workflows [55].

### Engineered tumor microenvironment models: sophisticated in vitro tumor simulators

While PDOs and tumor spheroids provide foundational 3D architectures, they often represent a simplified, epithelial-centric view of cancer. The tumor microenvironment is a dynamic, multi-cellular ecosystem that critically shapes malignancy, treatment response, and resistance. The next frontier in 3D modeling is the intentional and controlled engineering of this TME in vitro, moving from monotypic cultures to complex, interactive systems. This engineering occurs at three primary and often converging levels: through direct co-culture with stromal components, via the microphysiological control offered by organ-on-a-chip platforms, and through the spatial precision of 3D bioprinting.

Stromal co-culture describes the most direct method to engineer a TME in which stromal cells are reintroduced into cultures of cancer organoids or spheroids, creating a more physiologically relevant “tumor-stroma unit” for studying paracrine signaling and physical interactions [63]. Co-culturing tumor cells with cancer-associated fibroblasts recapitulates critical bidirectional crosstalk. CAFs deposit and remodel a dense, cross-linked extracellular matrix, creating a physical barrier that impedes drug penetration and promotes an invasive phenotype in cancer cells through mechanotransduction [64]. Furthermore, CAFs secrete a plethora of growth factors, cytokines, microRNAs, and other secreted proteins such as OPN/SPP1 that provide pro-survival, pro-stemness and pro-metastasis signals, directly inducing therapy resistance [65–68]. These models are essential for testing stroma-targeting agents and understanding how the stroma evolves under therapeutic pressure. Integrating immune cells is arguably the most crucial and challenging step for modeling modern immuno-oncology. Co-culture systems now allow for the study of tumor-immune interactions in a 3D context. PDOs can be co-cultured with autologous tumor-infiltrating lymphocytes to model cytotoxic T-cell killing, T-cell exhaustion, and the effects of immune checkpoint inhibitors [69–72]. Beyond autologous settings, PDOs can also be co-cultured with allogeneic immune cells, including engineered T cells or CAR-T products. In such co-cultures, PDOs are typically dissociated into single cells or small clusters and then re-aggregated with allogeneic immune effectors in a 3D matrix such as collagen or basement membrane extract, followed by live imaging or flow cytometry-based readouts of target cell killing, immune cell infiltration, and activation status [73]. This approach is particularly valuable when autologous tumor-infiltrating lymphocytes are unavailable, for example, in patients with low-immunogenicity tumors or those who have not yet undergone surgical resection, or when screening off-the-shelf cell therapy products where donor-recipient HLA matching is not required. These allogeneic systems thus provide a complementary platform for preclinical evaluation of cell-based immunotherapies. Similarly, incorporating tumor-associated macrophages can reveal how they are educated to adopt a pro-tumorigenic, immunosuppressive M2-like phenotype [74–76]. These systems are vital for screening novel immunotherapies and understanding mechanisms of immune escape. While creating perfusable vasculature remains a major goal, co-culturing with endothelial cells can initiate the formation of primitive vessel-like structures, mimicking the angiogenic tumor niche and providing a model for studying anti-angiogenic therapies and the crucial process of intravasation during metastasis [77–79]. The functional importance of incorporating vasculature into 3D cancer models has been recently demonstrated in small cell lung cancer (SCLC), where vascularized organoid models revealed a previously unrecognized mechanism of anti-tumor immunity. Campisi and colleagues showed that STING activation in tumor-associated endothelial cells facilitates NK cell recruitment and activation, leading to enhanced immune-mediated tumor cell killing. This vascular-dependent immune mechanism was entirely absent in non-vascularized tumor organoid cultures or conventional 2D systems, underscoring that certain aspects of tumor-immune interactions, particularly those involving endothelial cell signaling and immune cell trafficking, can only be faithfully modeled when a functional vasculature is present [80].

Beyond immune-vascular interactions, it is important to distinguish between different levels of vascular complexity achieved in current 3D cancer models, as this distinction has direct implications for drug delivery studies and tumor-vascular interaction modeling. Self-assembled, non-perfusable networks arise spontaneously when endothelial cells are co-cultured with cancer cells or fibroblasts in matrix-rich environments. These structures recapitulate certain angiogenic features such as endothelial sprouting, tube formation, and pericyte recruitment, which are valuable for studying early vascular morphogenesis and anti-angiogenic drug effects. However, they typically lack open lumens and functional perfusion, meaning they do not support convective drug transport or circulating cell interactions, which limits their utility for pharmacokinetic or metastasis modeling [81]. In contrast, engineered, lumenized, and perfusable vascular structures are achieved through advanced fabrication techniques such as microfluidic channel design, sacrificial bioprinting, or guided self-assembly in perfusable chips. These systems contain open, endothelium-lined lumens that support continuous fluid flow, enabling quantitative studies of drug extravasation, shear stress effects on cancer cell behavior, and circulating tumor cell adhesion and transendothelial migration [81, 82]. For example, microfluidic tumor-vascular chips have been used to demonstrate that flow-derived shear stress modulates endothelial cell orientation and polarization, as well as tumor spheroid expansion, while also revealing that perfusion significantly increases tumor cell proliferation and alters drug sensitivity compared to static conditions [81, 83]. Similarly, perfusable vascular networks have enabled real-time tracking of targeted liposome delivery into tumor compartments, providing a platform for evaluating vascular-targeted therapies [84]. Recognizing this spectrum of vascular complexity from non-perfused networks to fully perfused lumenized vessels is essential for selecting the appropriate model system for specific research questions, particularly when the goal is to study drug delivery or hematogenous metastasis [85]. The major challenge in co-culture is maintaining the viability and correct phenotypic state of all cell types over time, as their optimal culture conditions often conflict, a hurdle addressed by advanced media formulations and spatially defined co-culture setups.

Organ-on-a-chip (OOC) technology applies microfluidic engineering to create miniature, dynamic tissue cultures within continuously perfused microchambers [86]. For cancer modeling, OOC platforms move beyond static 3D culture by introducing crucial physiological forces and spatial organization [87]. Perfusion mimics blood and interstitial flow, which governs the delivery of nutrients, oxygen, and drugs, as well as the removal of waste, while shear stress influences endothelial and cancer cell signaling. A key strength is the ability to model multicellular tissue-tissue interfaces [88]. A canonical “metastasis-on-a-chip” device features a primary tumor chamber connected via a microfluidic channel lined with endothelial cells to a secondary chamber representing a distant organ, allowing for the real-time observation of the metastatic cascade: local invasion, intravasation, circulation, adhesion, extravasation, and colonization [89, 90]. Similarly, chips can model the blood-brain barrier for studying brain metastasis [91, 92]. Some advanced chips incorporate flexible membranes or matrices, allowing researchers to apply and measure cyclic mechanical strains mimicking breathing or peristalsis, or to study how dynamic matrix stiffness influences tumor progression [93, 94]. These systems provide an unprecedented level of control over the biophysical and biochemical microenvironment but often require specialized fabrication and operation expertise, and throughput is typically lower than that of standard multi-well plates.

3D bioprinting represents the apex of engineered control, using automated deposition to position living cells and biomaterials with precise spatial patterning [95]. In cancer research, this moves from allowing self-organization to prescribing organization. It can construct complex tumor geometries with defined size, shape, and internal architecture, invaluable for standardizing models for drug testing [96, 97]. The most powerful application is the spatial patterning of the TME. Researchers can design and print a tumor with concentric rings: a core of cancer cells, surrounded by a shell of CAFs in a stiff matrix, with an outer ring of endothelial cells. This level of control allows for the systematic study of how stromal cell arrangement influences cancer cell behavior in a way mixed co-cultures cannot [98]. Robotic bioprinters can also rapidly array hundreds of miniatures, reproducible tumor constructs into multi-well plates, paving the way for a new generation of high-throughput 3D screening platforms [99]. Furthermore, the concept of “bioprinting a patient’s tumor” is emerging, where cells from a biopsy are expanded, mixed into a bioink, and printed to create a personalized, architecturally defined model for therapy testing [100, 101]. For example, 3D bioprinted patient-derived cervical cancer organoids were established to demonstrate that NSUN6-mediated NDRG1 mRNA stabilization underlies radioresistance in cervical cancer [102]. Current limitations include the availability of truly biomimetic bioinks, potential shear stress damage to cells during printing, and challenges in maintaining long-term viability of complex tissues. Nevertheless, with the development of additive manufacturing technologies, 3D bioprinting is rapidly evolving into 4D, 5D and 6D versions for deconstructing and reconstructing the TME with engineer’s precision [103, 104]. These initial applications, while powerful, represent only the early stages of what bioprinting can offer; recent advances have further expanded its capabilities in vascularization, perfusion, and personalized modeling, as discussed below.

The unique capabilities of bioprinting have been increasingly recognized in recent years as essential for bridging the gap between simple 3D cultures and physiologically relevant tumor models. A systematic review of tumoroid technologies has highlighted bioprinting, together with microfluidic devices, as a key enabling technology for high-throughput personalized medicine applications, allowing for the precise spatial arrangement of multiple cell types and extracellular matrix components in a reproducible manner [105]. One of the most significant advantages of bioprinting over other 3D models is its ability to generate perfusable, vascularized tumor tissues. For instance, a bioprinted neuroblastoma model incorporating patient-derived tumor spheroids, endothelial cells, and mesenchymal stem cells in a gelatin-methacrylate/fibrin matrix demonstrated spontaneous micro-vessel formation and tumor cell invasion toward the vascular compartment, recapitulating early steps of metastatic dissemination in a manner not possible with non-vascularized spheroids [106]. Similarly, the integration of bioprinting with microfluidic chip technology has enabled the fabrication of self-assembled vascularized tumor arrays, where a vascular endothelial barrier surrounding breast cancer spheroids allows for controlled perfusion and diffusivity, providing a platform for studying drug delivery through the endothelial barrier [107]. Beyond vascularization, bioprinting has proven particularly valuable for preserving patient-specific tumor characteristics in personalized medicine applications. A printed gastric cancer model using patient-derived tumor chunks in tissue-specific bioinks retained original tumor characteristics and enabled rapid drug evaluation within two weeks of isolation, with drug response-related gene profiles showing remarkable similarity to patient tissues [108]. These advances underscore that bioprinting is not merely an incremental improvement but a transformative technology that uniquely enables the controlled integration of vascular, stromal, and tumor compartments, a level of architectural and compositional control that distinguishes it from self-assembled organoid and spheroid systems.

In conclusion, the engineering of the tumor microenvironment through co-culture, organ-on-a-chip, and bioprinting technologies is transforming 3D cancer models into sophisticated in vitro “tumor simulators.” Each approach offers a unique trade-off between biological complexity, physiological relevance, experimental control, and scalability. The integration of these technologies, for example, bioprinting a structured tumor-stroma construct and then perfusing it in an organ-chip device, represents the cutting edge, promising to deliver models that finally capture the full, dynamic complexity of human cancer. The key characteristics of the major 3D cancer platforms discussed above are compared in Table 1.

**Table 1:** Comparison of key 3D cancer model systems

Model System	Biological Fidelity	Microenvironment Control	Scalability	Throughput	Best Suited For
Tumor spheroids	Low-moderate (cell line dependent; lacks patient-specific genetics)	Low (only spontaneous gradients; no immune/stromal cells)	High	High (HTS compatible)	Mechanistic studies (gradients, penetration), first-pass 3D drug screening
Patient-derived organoids (PDOs)	High (preserves patient-specific genetics, histopathology)	Low-moderate (epithelial only; lacks native stroma and vasculature)	Low-moderate	Low-moderate	Personalized medicine, subtype-specific biology, resistance modeling
Stromal/immune co-culture	Moderate-high (adds TME components)	Moderate (controlled addition of specific cell types)	Moderate	Moderate	TME crosstalk, immunotherapy testing, stroma-mediated resistance
Organ-on-a-chip	Moderate-high (incorporates flow, shear, tissue interfaces)	High (perfusion, multi-compartment, mechanical forces)	Low-moderate	Low	Metastasis modeling, pharmacokinetics, multi-organ interactions
3D bioprinting	Moderate-high (spatially organized TME)	Very high (prescribed architecture, vascular channels possible)	Low (currently)	Low	Architectural studies, standardized constructs, complex TME reconstitution

### Model characterization and validation: from morphology to functional correlation

The increasing complexity and translational ambitions of 3D cancer models necessitate a rigorous and multi-parametric framework for their characterization and validation. A model is only as valuable as its ability to faithfully represent the biological reality it seeks to mimic. Therefore, comprehensive validation must answer a critical question: To what extent does the in vitro construct recapitulate the key molecular, cellular, architectural, and most importantly, functional properties of the human tumor? This validation process occurs across multiple, complementary tiers, forming an essential bridge between model development and confident biological or clinical inference.

The most immediate and visually compelling form of validation is histopathological and architectural fidelity. For patient-derived models, this involves direct comparison with the original clinical specimen. Standard hematoxylin and eosin staining of paraffin-embedded sections assesses whether the model retains tissue-specific architecture such as glandular lumens or papillary structures. Immunohistochemistry staining for lineage-specific markers, differentiation markers, proliferation indices, and key oncoproteins verifies the preservation of phenotypic states. Advanced 3D imaging modalities, including live-cell confocal microscopy and light-sheet microscopy, provide a volumetric understanding of model structure, revealing spatial distribution of cell types, necrotic cores, and invasive protrusions [109–112]. Phenotypic fidelity is best interpreted in the context of molecular and genomic consistency. For PDOs, targeted or whole-exome sequencing is mandatory to confirm the retention of pathognomonic driver mutations, copy number alterations, and structural variants, and to monitor genomic stability over passages [113]. Bulk RNA sequencing compares the global gene expression signature of the model to its tissue of origin and relevant clinical cohorts, confirming subtype classification and key oncogenic pathway activity [114]. Single-cell RNA sequencing offers a higher-resolution view, capable of identifying whether the model preserves the original tumor’s cellular heterogeneity and distinct subpopulations [20]. While molecular and morphological concordance is necessary, the most persuasive validation is functional. The gold-standard functional validation for a PDO is a direct comparison of its ex vivo drug sensitivity profile with the clinical outcome of the donor patient. Prospective and retrospective studies have shown that PDOs can predict patient response to chemotherapy, targeted therapy, immunotherapy and combinational regimen with significant accuracy, with a high positive predictive value for resistance being particularly powerful [26, 115, 116]. Validating that a model appropriately responds to specific molecular perturbations is also key, such as demonstrating expected sensitivity to a targeted agent in a model harboring the cognate mutation [117]. Furthermore, the model should exhibit hallmark behaviors of the cancer type it represents, such as the production of specific secreted factors or the ability to undergo relevant differentiation programs. As 3D models move towards clinical and industrial applications, standardization, reproducibility, and quality control become paramount [118]. The field must converge on a set of minimum reporting standards for different model types, including metrics like size distribution, viability threshold, and core molecular verification checks. A major challenge is variability introduced by reagents, protocols, and operator technique, making ring studies essential to assess inter-laboratory reproducibility. Moving away from subjective assessments is crucial, necessitating the development of automated, quantitative readouts such as high-content imaging analysis pipelines that quantify organoid morphology and standardized, matrix-appropriate viability assays.

Beyond traditional histopathological and molecular characterization, the field has witnessed a surge in computational approaches for automated phenotyping and predictive response profiling of 3D cancer models. Beginning around 2017, computer-aided image analysis tools have been developed to quantify organoid and spheroid morphology, including size, shape, circularity, bud formation, and internal heterogeneity, from brightfield and fluorescence microscopy images in a high-throughput manner. Early work demonstrated that convolutional neural networks (CNNs) could predict tissue differentiation in organoids based solely on brightfield images, outperforming human experts [119]. This paradigm has since been substantially advanced by the development of user-friendly deep learning platforms specifically designed for organoid and spheroid analysis. For instance, OrganoID provides robust automated recognition, labeling, and tracking of single organoids with 95% agreement with manual measurements of organoid count and 97% for size, without requiring parameter adjustments across different tumor types [120]. Similarly, SpheroScan leverages Mask R-CNN for automated spheroid image detection and segmentation in a web-based, user-friendly format [121]. More recently, deep learning frameworks have been extended to analyze organoid-immune cell co-cultures, enabling real-time monitoring of immunotherapy responses in patient-derived pancreatic cancer organoids [122]. Critically, pre-treatment morphological features extracted from 3D models using these computational tools have been shown to predict subsequent therapeutic response, with aggregated morphological indicators derived from deep learning-based segmentation correlating strongly (correlation coefficient > 90%) with standard ATP-based viability assays [123]. As these computational tools continue to mature with the adoption of vision transformers and multimodal foundation models, they are poised to transform model characterization from a manual, descriptive process into an automated, quantitative, and predictive discipline, significantly enhancing the scalability and translational power of 3D cancer platforms.

In conclusion, the characterization and validation of 3D cancer models is an ongoing, multi-layered process integral to their scientific utility, with emerging computational approaches for automated phenotyping and predictive response characterization further expanding their translational potential. It requires a holistic approach that integrates traditional pathology with modern omics technologies and anchors itself in clinically relevant functional outputs. Rigorous validation transforms a complex cellular aggregate from a biological curiosity into a credible, reproducible, and predictive experimental platform, laying the foundation for these models to fulfill their promise in de-risking drug development and personalizing cancer therapy.

## Applications of 3D cancer models in precision oncology and drug development

**Fig. 2:**
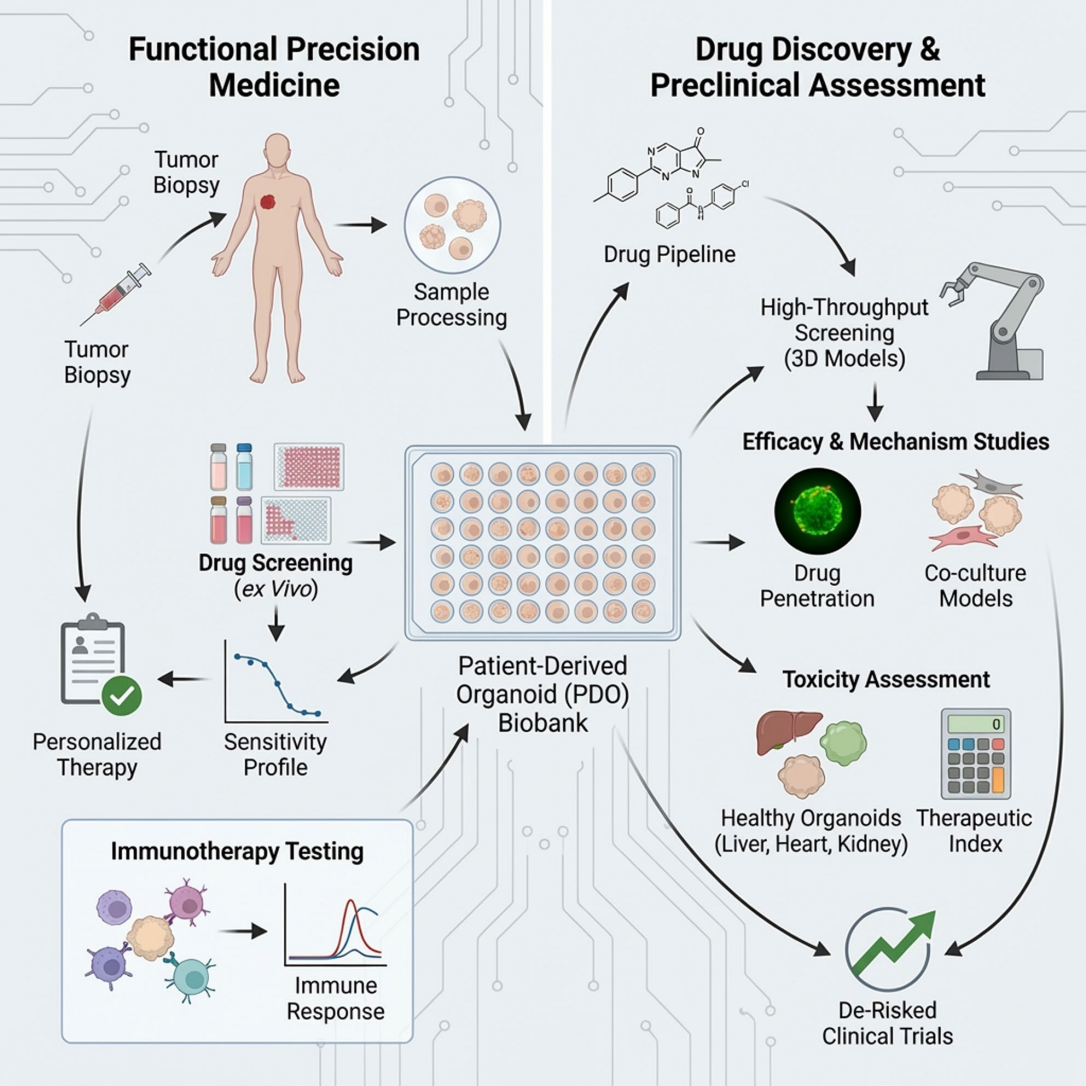
Applications of 3D cancer models in precision oncology and drug development. Integration of PDOs and related 3D platforms across clinical decision-making and the therapeutic development pipeline. In functional precision medicine, tumor biopsies are processed to generate PDO biobanks that enable rapid ex vivo drug testing and the derivation of patient-specific sensitivity profiles, informing personalized treatment strategies. In drug discovery, 3D models are incorporated from early high-throughput screening to mechanistic validation, providing physiologically relevant contexts for assessing compound penetration, efficacy and resistance mechanisms. Engineered co-culture and immune-integrated systems further capture tumor-stroma and tumor-immune interactions, whereas healthy organoid platforms support early toxicity evaluation and therapeutic index estimation. Collectively, these applications position 3D cancer models as translational intermediates that enhance predictive power, guide biomarker development and de-risk clinical trial design

The true value of any model system is measured by its ability to generate actionable insights and improve outcomes. 3D cancer models, particularly PDOs, are transitioning from research tools to integral components of the translational oncology pipeline. They offer a dynamic, human-relevant platform to address two of the field’s most pressing challenges: the implementation of truly personalized therapy and the acceleration of effective drug discovery. This section details how these models are being deployed to guide clinical decisions in real-time, to serve as high-fidelity avatars for in vitro drug screening, and to reshape the preclinical assessment of novel therapeutic agents, ultimately aiming to bridge the costly and inefficient gap between bench and bedside (Fig. 2).

### Functional precision medicine: PDOs as clinical decision support tools

Precision oncology has historically been driven by genomic profiling, matching identified mutations to targeted therapies. However, genomic predictors often fail due to pathway redundancy, adaptive resistance, and the complex influence of the tumor microenvironment [124]. Functional precision medicine complements genomics by directly testing drug efficacy on a living model of the patient’s tumor [125]. This approach, which ranges from PDO-based drug testing to ex vivo sensitivity assays on fresh tumor specimens, offers a functional readout of drug vulnerability that integrates genetic and non-genetic determinants of response, complementing static genomic profiling [126]. Here, PDO biobanks emerge as a powerful clinical decision-support platform, aiming to predict patient-specific therapeutic responses ex vivo.

The proposed clinical pipeline involves establishing a living PDO biobank from a patient’s diagnostic or surgical sample in parallel with standard pathological and genomic workup. These organoids are then subjected to a panel of drug treatments relevant to the patient’s cancer type and genomic profile. Quantitative sensitivity profiles are generated within a clinically actionable timeframe (typically 2–4 weeks) using viability or apoptosis readouts from high-throughput imaging or ATP-based assays [127, 128]. Numerous studies across cancer types, including colorectal, pancreatic, breast, gastric, sarcoma, lung, ovarian, and many others, have demonstrated strong correlations between PDO drug sensitivity and the corresponding patient’s clinical outcome [129–135]. For instance, a drug sensitivity statistic obtained from advanced lung cancer PDOs was significantly associated with clinical response, with overall concordance rates reported in the range of approximately 70–80% [136]. A biliary tract cancer PDO platform reported that PDO-based chemotherapy screening results were consistent with the actual clinical responses in 92.3% of cases, supporting the clinical relevance of organoid drug sensitivity readouts in a liver-related cancer setting [137]. These findings underscore the potential utility of PDO platforms as functional tools for therapeutic stratification. Notably, PDO models show a high negative predictive value; resistance observed in PDOs robustly predicts treatment failure in the clinic [138, 139]. This predictive power extends beyond targeted therapies to include chemotherapies and, with the integration of immune cells, immunotherapies. Strikingly, in a metastatic gastrointestinal cancer model receiving targeted or chemotherapy, PDOs achieved a 100% negative predictive value [140]. Several pilot prospective clinical trials have now reinforced this correlation and, critically, demonstrated the feasibility of integrating PDO testing into real-world clinical pathways, addressing practical hurdles such as sample acquisition from small biopsies and achieving rapid turnaround times [141, 142]. In metastatic colorectal cancer, a phase 2 single-center study showed that PDO-based drug sensitivity testing was successfully implemented in a clinical setting, with results available within a clinically actionable timeframe and correlating with patient outcomes [143]. Similarly, a prospective feasibility study in relapsed or refractory pediatric cancers demonstrated that functional precision medicine using PDOs could be successfully integrated into clinical care, with drug response profiles generated for the majority of enrolled patients [144].

PDO-based functional testing offers distinct advantages over traditional static biomarkers. First, it captures the integrated effects of genetic and non-genetic determinants of drug response within a patient’s tumor. For example, it has been demonstrated that drug responses observed in PDOs were highly concordant with clinical outcomes, even in cases where genomic alterations alone could not fully explain treatment sensitivity or resistance [140]. Several case studies further illustrate the clinical utility of PDO-based functional testing. Yin and colleagues developed patient-derived tumor-like cell clusters (PTCs) from various cancer types and demonstrated that PTC-guided drug selection led to clinical responses in patients with refractory cancers, including complete remission in a case of advanced cholangiocarcinoma [145]. In hepatocellular carcinoma, PDO-based drug screening informed conversion therapy that resulted in a pathologic complete response, enabling subsequent curative resection [146]. Similarly, medium-throughput drug screening of PDOs from colorectal peritoneal metastases successfully identified active regimens that guided personalized therapy in patients who had exhausted standard treatment options [147]. Similarly, pharmacotyping of pancreatic cancer organoids revealed heterogeneous drug sensitivities that were not predictable solely from mutational profiling [148]. Second, PDO platforms enable the rapid empirical testing of drug combinations at different ratios and schedules. For instance, combinational targeting strategies was systematically evaluated in colorectal cancer organoids harboring KRAS pathway alterations, identifying synergistic interactions that would have been difficult to rationally design based on genomic data alone [149]. Such approaches provide a functional basis for tailoring personalized combination regimens within clinically relevant timeframes. Third, PDO testing can uncover unexpected therapeutic vulnerabilities beyond standard-of-care regimens. For example, a colorectal cancer organoid biobank was established to demonstrate that functional drug screening identified subtype-specific sensitivities that were not readily predictable from genomic alterations alone, revealing therapeutic opportunities that might otherwise have been overlooked [150]. Such findings highlight the potential of PDO platforms to identify off-label or non-standard treatment options for patients who have exhausted conventional therapies.

Despite its promise, significant challenges remain for broad clinical implementation [151]. The time-to-result must compete with the urgency of treating aggressive cancers. Researchers found that the turnaround time from biopsy to actionable PDO drug-response readouts often spans 4–10 weeks, which may be incompatible with the clinical urgency of aggressive cancers [152]. In addition, tumor heterogeneity means a single biopsy may not fully represent the entire disease landscape, especially in metastatic settings [153]. Direct comparison of drug responses in organoids derived from matched primary tumors and metastatic sites has revealed both concordant and discordant sensitivities, underscoring the need for multi-lesion sampling when feasible [154]. Despite these challenges, multiple case studies have demonstrated clinical benefit from PDO-guided therapy, including successful treatment of refractory cancers, pathologic complete response enabling curative resection, and identification of active regimens for patients who had exhausted standard options [145–147]. This fact implicates that a single-site biopsy used to generate a PDO may not fully recapitulate the spatial and clonal diversity of metastatic disease, potentially omitting resistant subclones present in other lesions, thereby limiting the generalizability of PDO-derived drug-response predictions. Furthermore, the cost and infrastructure required for robust, standardized PDO testing are substantial [155]. Future directions to overcome these hurdles involve streamlining culture protocols using automated platforms, developing strategies for deriving models from circulating tumor cells for serial monitoring of disease evolution, and establishing large, shared PDO biobanks linked to rich clinical annotation. Such biobanks will enable the use of artificial intelligence to mine sensitivity data across thousands of “virtual patients,” facilitating the discovery of new predictive biomarkers and the refinement of treatment algorithms beyond individual anecdotal matches.

Furthermore, an important caveat in interpreting PDO-based drug response data relates to sample origin. For aggressive tumor types including triple-negative breast cancer, high-grade serous ovarian cancer, pancreatic ductal adenocarcinoma, and relapsed or refractory malignancies, biopsies or surgical specimens are frequently obtained from patients who have already received systemic therapies, whether neoadjuvant, adjuvant, or palliative. Consequently, the resulting organoids may not represent treatment-naïve disease but rather therapy-exposed or treatment-selected tumor populations. Direct evidence for this concern comes from studies showing that organoids derived from post-treatment tissues exhibit altered drug sensitivity profiles compared to treatment-naïve counterparts. It has been demonstrated that gastric and colon cancer organoids constructed from patient tissues after chemotherapy showed significantly increased sensitivity to paclitaxel and 5-fluorouracil compared to freshly isolated tumor cells, accompanied by downregulation of stemness-related genes, suggesting that prior treatment history leaves a functional imprint on PDO drug responses [156]. In head and neck cancer, long-term expansion of organoids from treated patients has been shown to drive clonal evolution, with later passages exhibiting altered treatment responses compared to early passages, further complicating the interpretation of drug screening results [157]. More broadly, the limitations of patient-derived models in recapitulating the treatment-naïve tumor state have been recognized as a critical consideration for precision medicine applications [158]. These issues are particularly relevant when PDOs are proposed as predictive tools for first-line therapy selection, where treatment-naïve samples are ideal but often unavailable for aggressive cancers that require immediate treatment. Researchers should therefore document prior treatment history thoroughly and, when possible, obtain pre-treatment biopsies or utilize circulating tumor cell-derived organoids as a less invasive alternative for longitudinal sampling. Acknowledging this bias strengthens the translational robustness of PDO-based functional precision medicine.

### Drug discovery and preclinical assessment: from screening to de-risking translation

The drug development pipeline is notoriously inefficient, with high attrition rates due to lack of efficacy or unforeseen toxicity in clinical trials, a failure often rooted in the poor predictive value of conventional preclinical models. Comprehensive analyses of clinical trial success rates have shown that oncology drugs have the lowest likelihood of approval among all major therapeutic areas, with only 3–5% of candidates advancing from Phase I to regulatory approval [159, 160]. Among the primary reasons for failure, lack of efficacy accounts for approximately 52% and safety/toxicities for approximately 24% of phase II and III trial terminations, with oncology representing the therapeutic area with the highest proportion of failures [161]. The poor predictive value of conventional 2D cell line screens and PDX models is widely recognized as a major contributor to both categories of failure: 2D cultures generate false-positive efficacy signals that do not translate to patients, while PDX models fail to recapitulate human-specific toxicities and lack the human immune context necessary for evaluating immunotherapies. 3D cancer models are now being integrated across this pipeline to improve the quality of lead compounds, elucidate their mode of action, predict toxicity, and ultimately de-risk the transition to clinical trials.

In the early discovery phase, high-throughput screening is being revolutionized by 3D models. The integration of uniform tumor spheroids and increasingly scalable PDO biobanks introduces critical biological filters absent in 2D. Compounds must penetrate a multicellular structure and remain effective under physiological gradients of nutrients and oxygen, thereby weeding out artifacts of 2D-specific biology and improving the probability that identified “hits” will possess favorable pharmacokinetic properties [49, 162]. These models also facilitate additional phenotypic screens for outcomes such as disruption of spheroid integrity, inhibition of invasion into a surrounding matrix, or reversal of an immunosuppressive microenvironment in a co-culture setting [163–165]. Technologies like ultra-low attachment spheroid microplates and microfabricated microwell arrays enable the generation of thousands of uniform 3D constructs compatible with automated screening workflows [166, 167]. Microcavity arrays represent an advanced iteration of this approach, enabling high-throughput automated organoid formation from stem cell aggregates with exceptional uniformity and compatibility with robotic liquid handling [55]. Beyond plate-based formats, microfluidic platforms have greatly expanded the throughput and capabilities of 3D drug screening. Droplet-based microfluidics allows nanoliter-scale combinatorial drug testing on biopsy-derived spheroids and organoids, enabling thousands of unique conditions to be screened from limited clinical samples [168–170]. Patient-derived micro-organospheres generated in microfluidic droplets have been successfully used to predict clinical responses in cancer patients within clinically actionable timeframes [171]. Additionally, integrated microfluidic devices capable of generating self-established concentration gradients enable rapid drug screening of biopsy-derived spheroids with minimal sample consumption [172]. However, it is worth noting that ultra-low attachment microplate choice among different brands significantly influences spheroid formation, functionality, and assay reproducibility, underscoring the need for evidence-based standardization in 3D culture systems [173].

Once a lead compound is identified, 3D models provide a superior context for detailed efficacy and mechanism-of-action studies. Confocal microscopy of fluorescently tagged drugs in 3D structures allows for quantitative analysis of penetration kinetics and distribution heterogeneity, guiding medicinal chemistry efforts to optimize drug formulations [174, 175]. Furthermore, single-cell RNA sequencing of treated versus untreated organoids can reveal how different subpopulations (e.g., proliferative, stem-like) respond to therapy, identifying which clones are eradicated and which persist, offering early clues about potential resistance mechanisms [176]. Engineered co-culture models are particularly valuable for dissecting how stromal components modulate drug efficacy, enabling researchers to ask, for instance, whether the presence of cancer-associated fibroblasts confers resistance via secreted factors, thereby guiding the development of rational combination therapies from the earliest stages [177, 178].

A safe drug must selectively kill cancer cells while sparing healthy tissues. Here, 3D models also contribute to toxicity assessment and therapeutic index prediction. Healthy organoids derived from liver, heart, and kidney can be exposed to lead compounds to assess organ-specific cytotoxic effects [179]. For instance, it has been reported that organoid-based models respond to nephrotoxic compounds with reproducible structural and transcriptional changes that mirror known renal injury pathways, supporting their application in preclinical safety screening [180]. Co-culturing tumor organoids with these “off-target” organoids in a shared medium or multi-organ chip system enables an initial, human-cell–based estimation of on-target efficacy versus off-target toxicity [181]. For immunotherapies, models incorporating human immune components and normal tissue structures may help predict immune-related adverse reactions (irAEs), such as colitis or pneumonitis, prior to animal testing [182]. For example, a human intestinal organoid system incorporating tissue-resident immune components demonstrated that immune activation within co-culture framework induced epithelial inflammatory programs and cellular stress responses that resemble features observed in immune-related colitis [183]. Similarly, another recent study employed an organoid-immune co-culture system to demonstrate that ICI treatment induces T cell-derived TNF signaling, which activates MLCK1-dependent tight junction disruption and increases gut permeability. Pharmacological inhibition of MLCK1 preserved epithelial barrier integrity and mitigated colitis without impairing antitumor efficacy [184]. These findings illustrate how immune-integrated organoid models can help dissect mechanisms underlying immune-related colitis and identify strategies to reduce toxicity while maintaining therapeutic benefit.

The collective application of 3D models throughout the preclinical pipeline aims to build a more predictive data package for clinical entry. Demonstrating efficacy not just in cell lines but in a panel of genomically annotated PDOs provides stronger evidence of potential clinical activity across a heterogeneous patient population and helps prioritize which patient subsets are most likely to respond, thereby enriching clinical trial design. Mechanistic studies in these models can reveal predictive biomarkers of response or resistance early in development. While animal studies remain regulatory necessities, a robust, human-relevant dataset from 3D models can provide greater confidence in a compound’s potential, potentially reducing the scale and cost of animal testing and helping to halt the development of doomed candidates earlier in the process. In summary, the integration of 3D cancer models transforms the preclinical phase from a sequential checklist into an integrative, predictive science, increasing the probability that drugs entering clinical trials will be both effective and safe.

## Deciphering mechanisms of therapy resistance by using 3D cancer models

**Fig. 3:**
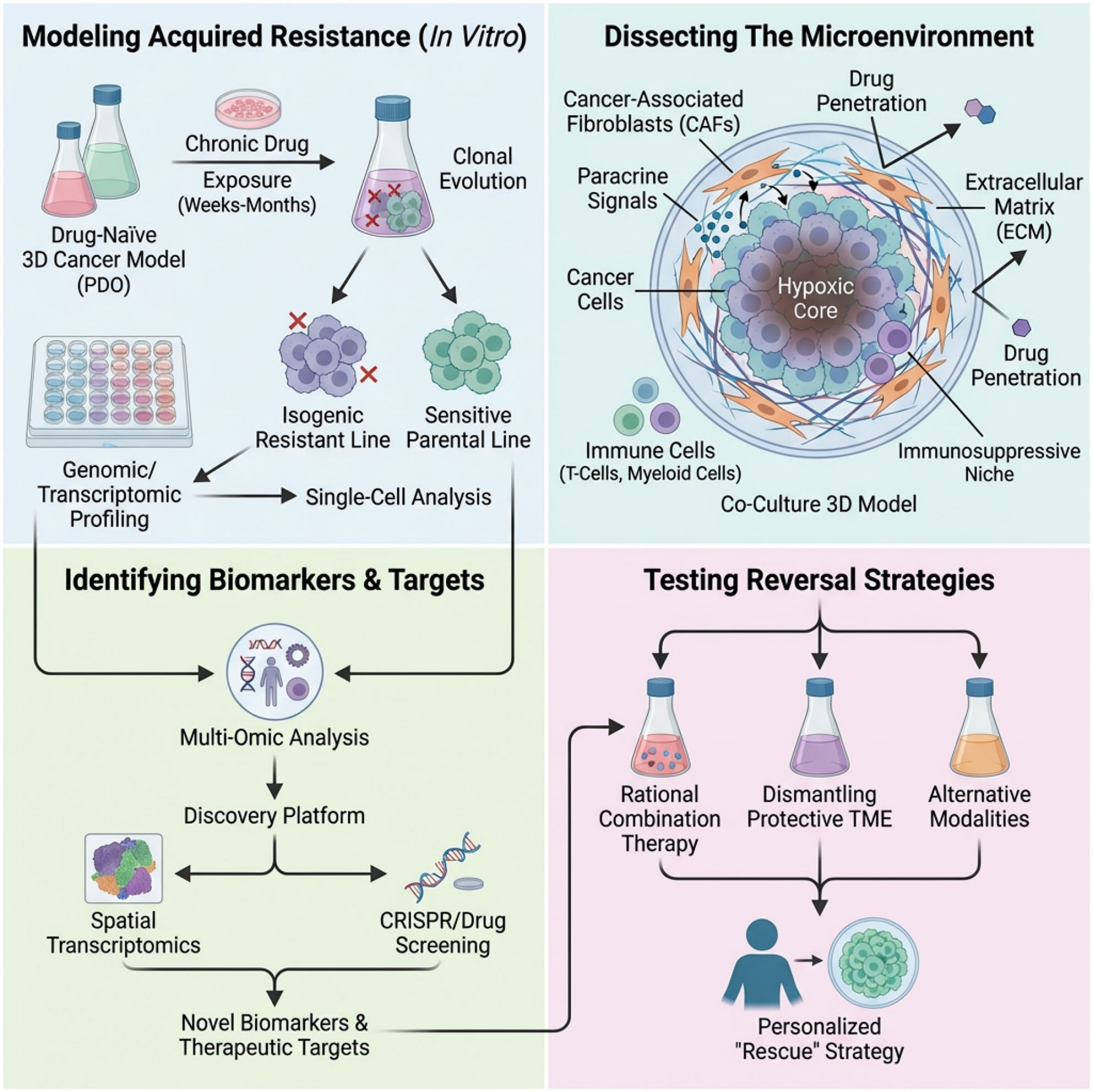
Leveraging 3D cancer models to decipher and overcome therapeutic resistance. Experimental framework illustrating how 3D cancer platforms are used to model acquired resistance, dissect microenvironmental protection, and develop reversal strategies. Chronic drug exposure of initially sensitive PDOs enables in vitro clonal evolution and the generation of isogenic resistant derivatives, which can be interrogated by genomic, transcriptomic and single-cell analyses to define resistance-associated alterations. Engineered co-culture systems incorporating cancer-associated fibroblasts, immune cells and extracellular matrix components recapitulate hypoxic, immunosuppressive and drug-limiting niches, allowing mechanistic dissection of stromal shielding and paracrine survival signaling. Integrated multi-omic and functional screening approaches identify candidate biomarkers and actionable dependencies, supported by spatial profiling and CRISPR or pharmacological perturbation. These insights are subsequently translated into rational combination therapies, microenvironment-targeting strategies and alternative treatment modalities, positioning 3D models as patient-relevant platforms for personalized resistance rescue and therapeutic re-sensitization

Therapeutic resistance, whether intrinsic or acquired, remains the ultimate barrier to curing cancer. It is a dynamic, adaptive process driven by the genetic evolution of cancer cells and their reciprocal interactions with a protective tumor microenvironment. Traditional models have struggled to capture this spatial and temporal complexity. 3D cancer models, with their capacity for long-term culture, controlled perturbation, and multicellular engineering, now provide a powerful experimental arena to induce, observe, and dissect resistance as it unfolds. This section explores how these models are being leveraged to model the evolution of resistant clones, to deconstruct the microenvironment’s role as a sanctuary, and to functionally identify and test strategies to overcome or prevent resistance (Fig. 3).

### Modeling acquired resistance by tracking clonal evolution in vitro

A key advantage of PDOs and other expandable 3D cultures is their suitability for long-term, iterative experiments that mirror the chronic drug exposure encountered in vivo. This enables researchers to directly model the process of acquired resistance and study its underlying mechanisms in a controlled, tractable setting. The standard experimental paradigm involves exposing drug-naïve, sensitive PDOs or other 3D cultures to a sub-lethal or gradually increasing concentration of a therapeutic agent over several weeks to months. This sustained selective pressure enriches for pre-existing minor resistant subclones or induces adaptive cellular changes, culminating in the outgrowth of a stably resistant population. The resulting isogenic resistant line can then be compared directly to its parental, sensitive counterpart, which is a powerful paired system rarely available from clinical samples but essential for pinpointing causal changes. This in vitro evolution approach allows for the systematic uncovering of both genetic and non-genetic mechanisms. Genomic sequencing of the resistant derivative can identify the acquisition of secondary mutations, copy number alterations, or amplifications of bypass pathways that confer resistance. For example, analysis of EGFR-mutant lung cancer PDOs following exposure to targeted therapy demonstrated that resistant derivatives acquired genomic alterations, including copy number gains and activation of alternative signaling pathways, consistent with clinically observed bypass resistance; comparative genomic profiling between drug-naïve and resistant organoids revealed clonal evolution under therapeutic pressure, supporting the use of PDO-based sequencing to identify secondary resistance events [185]. Crucially, this process is equally adept at revealing non-genetic adaptive states, such as a reversible drug-tolerant persister (DTP) phenotype. Transcriptomic and epigenomic profiling of these models can uncover transient but stable resistance programs, like an epithelial-to-mesenchymal transition signature or a shift toward a slow-cycling, stem-like state that is intrinsically less vulnerable to therapy. This point is supported by the findings observed in a colorectal cancer PDO platform, where chemotherapy-resistant MEX3A positive cells were shown to enter a transient, fetal-like progenitor state rather than acquiring stable genetic resistance; these DTP cells suppressed canonical stem-cell signaling immediately after treatment and later reinitiated tumor growth, suggesting that relapse can arise from reversible adaptive reprogramming rather than new mutations [186]. In addition, by initiating resistance induction experiments from a heterogeneous PDO population, researchers can also study the role of tumor heterogeneity in resistance, using single-cell lineage tracing and sequencing technologies to help distinguish whether resistance is driven by the outgrowth of a dominant pre-existing clone versus the selection of distinct subclones under different selective pressures. A recent study barcoded heterogeneous patient-derived colorectal cancer organoids and tracked clonal dynamics under sequential drug treatments using single-cell multi-omics, showing that certain targeted therapies promoted the outgrowth of specific pre-existing subclones, whereas other treatments induced adaptation with limited changes in clonal structure, indicating a more plasticity-driven response rather than dominance of a single clone [187]. Once a resistant PDO model is established, it can be leveraged for functional re-sensitization screens, in which compound libraries are tested to identify agents that restore sensitivity to the original therapy and thereby nominate rational combination strategies. Such an approach has been applied in CRC PDOs, where drug-repurposing libraries were screened to identify re-sensitizing compounds, and epigenetic modulators have been shown to re-sensitize platinum-resistant tumor-derived organoids to carboplatin [188].

### Dissecting the role of TME in protection and resistance

While cancer cell-intrinsic mutations are critical, a substantial body of evidence implicates the tumor microenvironment as an active architect of therapy resistance, providing biochemical sanctuaries and physical barriers. 3D models, particularly those engineered with stromal components, offer an unparalleled opportunity to deconstruct this “stromal shield” and establish causality in a controlled, human-relevant system.

Cancer-associated fibroblasts are master regulators of the protective niche. Co-culturing tumor organoids or spheroids with CAFs can render cancer cells resistant to chemotherapy, targeted therapy, and radiation [189–191]. Using transwell systems to separate cells, researchers can demonstrate that this protection is largely mediated by paracrine signaling, and subsequent cytokine profiling can identify responsible factors like hepatocyte growth factor which activates MET signaling to bypass EGFR inhibition, or interleukin-6 which activates pro-survival STAT3 pathways [192, 193]. Beyond soluble factors, CAFs deposit and cross-link a dense, stiffened extracellular matrix [194]. In 3D models, this remodeled environment physically impedes drug penetration, a phenomenon quantifiable using imaging techniques like fluorescence recovery after photobleaching [195, 196]. The increased matrix stiffness itself also activates integrin-mediated mechanotransduction pathways in cancer cells, promoting survival and stemness [197]. Furthermore, CAFs can undergo metabolic reprogramming and secrete metabolites like lactate that cancer cells can utilize, which represents a symbiotic coupling that can be mapped in co-culture models using stable isotope tracing to identify metabolic vulnerabilities [198, 199].

Modeling the immune microenvironment is central to understanding resistance to immunotherapies. Co-culturing PDOs with autologous tumor-infiltrating lymphocytes or engineered T-cells allows for the real-time study of T-cell function, migration, and exhaustion [200]. Flow cytometry of recovered T-cells can reveal the upregulation of checkpoint markers like PD-1 and a loss of effector cytokines, and the system can be used to test the rescue effect of checkpoint blockade directly on the patient’s own immune-tumor unit [201, 202]. Incorporating immunosuppressive myeloid cells including M2-polarized macrophages or MDSCs into organoid/spheroid cultures can recreate an immunosuppressive niche and enables functional testing of agents aimed at re-educating or depleting these populations, such as CSF1R-directed macrophage-modulating strategies in glioblastoma PDO models [203]. Additionally, immune and stromal cells secrete a milieu of cytokines that can directly promote cancer cell survival; for example, IL-17 produced in the TME has been shown to promote resistance in colorectal cancer models, and its neutralization can be tested as a sensitizing strategy [204].

The dysfunctional tumor vasculature creates regions of chronic hypoxia, another potent driver of resistance. Spheroids and organoids naturally develop oxygen gradients, and the hypoxic core can be profiled to reveal the upregulation of HIF-1α target genes that promote angiogenesis, metabolic switching, and increased DNA repair capacity, all of which contribute to treatment failure [205]. For example, in pancreatic ductal adenocarcinoma PDO models, hypoxic and acidic stress was shown to promote resistance to gemcitabine plus nab-paclitaxel through HIF-1α-dependent upregulation of AVL9, while pharmacologic targeting of AVL9 re-sensitized PDO models to chemotherapy [206]. Treating 3D models in controlled hypoxia chambers can directly link low oxygen tension to these specific resistance pathways [207]. Beyond relying on spontaneously formed hypoxic cores, controlled hypoxia chambers provide a technically robust platform to interrogate oxygen-dependent resistance mechanisms in 3D models. In one such approach, a multi-sample hypoxia chamber system was developed which enables organoids to be cultured and treated under precisely defined oxygen tensions, allowing direct comparison of therapeutic responses under normoxic versus hypoxic conditions [208]. Such systems make it possible to causally link reduced oxygen tension to activation of HIF-dependent transcriptional programs and altered radiosensitivity or chemosensitivity, thereby strengthening mechanistic inferences beyond correlative observations in spontaneously hypoxic spheroids.

In addition to hypoxia, three-dimensional tumor models can also recapitulate extracellular acidosis, creating spatially restricted acidic niches that are experimentally tractable. In colorectal tumor spheroids, acid-exposed cells can be separated from hypoxic populations and transcriptionally profiled, demonstrating that acidic and hypoxic compartments are distinct and can be interrogated as separate microenvironmental states [209]. In pancreatic ductal adenocarcinoma, acid adaptation was shown to promote organoid growth and to increase drug resistance, providing a direct example of how sustained extracellular acidosis can drive a therapy-tolerant phenotype in patient-relevant 3D models [210]. Mechanistically, recent work has begun to connect acidosis to specific stress-response pathways that are relevant to treatment failure. A representative example is provided by a study combined pH-dependent sorting of acid-exposed cancer cells from 3D colorectal spheroids with transcriptomic profiling and reported consistent hallmarks of the acidic state, which revealed actionable vulnerabilities to ATM/ATR inhibitors in acid-conditioned cells [211]. Together, these studies illustrate how acidic microenvironments in 3D models can be isolated and profiled to link low pH to concrete resistance-associated programs and to nominate rational sensitization strategies.

### Identifying novel resistance biomarkers and targets

A primary translational goal of modeling resistance in vitro is to move beyond mechanistic understanding toward the identification of predictive biomarkers and actionable therapeutic targets. The unique experimental access afforded by isogenic sensitive or resistant 3D model pairs creates a powerful discovery platform in which molecular alterations can be systematically compared to define resistance-associated signatures that may serve as biomarkers, while concurrently revealing druggable dependencies that represent candidate targets.

The most straightforward strategy involves multi-omic profiling of paired 3D models to generate candidate resistance biomarkers. In PDOs, proteotranscriptomic profiling such as RNA-seq plus quantitative proteomics has linked baseline molecular programs to differential drug response, enabling the nomination of predictive transcriptional and protein-level biomarkers [212]. Integrating regulatory and epigenetic layers such as ATAC-seq with drug screening across PDO biobanks further refines these biomarkers by uncovering resistance-associated regulatory programs not evident from DNA sequencing alone [213]. Complementary phosphoproteomic analyses identify activated kinase nodes and signaling rewiring events that not only function as dynamic biomarkers of resistant signaling states but also expose therapeutically actionable kinase targets [214]. To resolve heterogeneity that obscures clinically relevant subpopulations, spatial and single-cell technologies enable the identification of location-defined and cell-state-specific biomarkers. Spatial transcriptomics applied to sectioned 3D models can delineate resistance niches characterized by stromal or vascular cues [215], while single-cell RNA sequencing before and after treatment pinpoints rare persister populations that survive therapy, yielding gene signatures of early resilience that can be mined for surface biomarkers or cell-state-specific drug targets [216–218]. Once resistance-associated states are molecularly defined, 3D models provide a functional platform for target validation. CRISPR-based genetic screens performed in resistant organoid systems can identify synthetic lethal interactions specific to the resistant state, directly nominating candidate therapeutic targets [219]. Likewise, high-throughput pharmacological profiling can uncover newly acquired drug dependencies, informing rational target prioritization and therapeutic switching strategies [220]. Finally, translation requires that candidate biomarkers be validated in annotated patient cohorts with treatment outcomes, and that prioritized targets demonstrate efficacy in patient-derived 3D models before clinical advancement [221].

### Testing strategies to reverse or circumvent resistance

The ultimate objective of modeling resistance is not merely to understand it, but to overcome it. 3D cancer models provide a critical, patient-specific testing ground for evaluating therapeutic strategies designed to prevent, reverse, or bypass resistance, enabling rapid iteration from mechanistic insight to actionable clinical hypotheses.

Once a resistance mechanism is elucidated, it directly informs the design of rational combination therapy. If resistance is driven by activation of a compensatory pathway, PDO models can be used to test combinations of the original agent with inhibitors of the bypass pathway. For example, phosphoproteomics-guided combinations have been evaluated in PDOs to match activated kinases with inhibitor cocktails and quantify synergy in dose-response matrices [214]. For genetic resistance, such as secondary mutations emerging under targeted therapy, PDO systems enable functional evaluation of next-generation agents and novel combinations tailored to overcome specific alterations, illustrating the translational value of PDO-guided therapeutic design in precision oncology [222]. More proactively, treatment-naïve PDOs can be leveraged to evaluate whether initiating therapy with rational drug combinations from the outset suppresses the outgrowth of resistant subclones, which complements individualized treatment decision-making and demonstrates how PDO-based drug screens can serve as preclinical predictive biomarker platforms to inform clinical strategies [138].

Strategies aimed at dismantling the protective tumor microenvironment can be rigorously evaluated in engineered co-culture models. For example, CAF-integrated PDO platforms have been developed to recapitulate stromal support and enable direct testing of CAF-targeting or ECM-modulating interventions for their ability to restore drug sensitivity and, in matrix-rich settings, improve effective drug access [223]. Immune-oncology organoid co-cultures are likewise well suited for testing combination immunotherapies, including the concept of adding a TGF-β pathway inhibitor to checkpoint blockade to relieve immunosuppression and to evaluate functional rescue of T-cell activity in a patient-relevant setting [224–226]. In models incorporating endothelial cells such as tumor-vascular microfluidic co-cultures, anti-angiogenic agents can be assessed for their capacity to promote vascular normalization phenotypes and to improve drug delivery into the tumor compartment, providing a controllable platform to quantify delivery or response changes under defined perfusion conditions [227].

These models also facilitate the testing of alternative modalities and sequential strategies. The order of treatments can be critical, and 3D models have been used to compare outcomes across different drug schedules and combination regimens, including systematic optimization of multi-drug combinations in PDO platforms [228, 229]. The response to physical modalities like radiation can likewise be studied in the context of 3D oxygen control or gradients, including engineered systems that explicitly manipulate hypoxia while irradiating embedded spheroids or organoids to quantify oxygen-dependent radioresponse [230, 231]. Furthermore, PDOs can serve as avatars for optimizing autologous adoptive cell therapies, as tumor-organoid co-cultures have been used to functionally assess CAR-T activity and compare constructs/conditions in a patient-relevant 3D context [232–234]. Aggregatively, these approaches point toward a future of personalized “rescue” trials, where a resistant PDO line from a progressing patient is screened against a panel of salvage regimens to empirically identify the most promising next-line option, an approach already being tested in prospective validation efforts and trial frameworks using PDO-guided treatment selection [116, 235].

## Current challenges and limitations

**Fig. 4:**
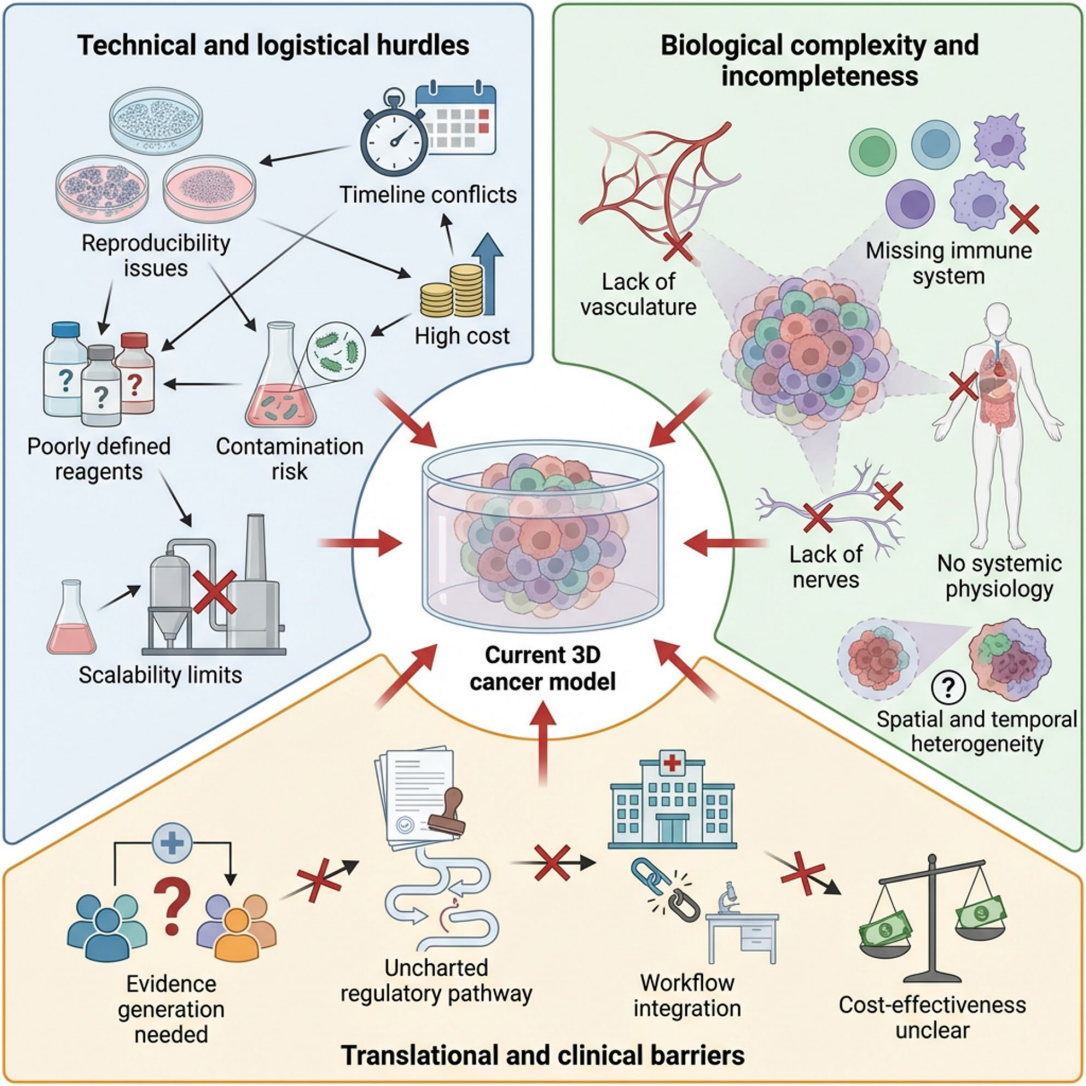
Current limitations and translational barriers of 3D cancer models. Key technical, biological and clinical challenges constraining the broader implementation of 3D cancer platforms. Technical and logistical hurdles include variability in establishment success rates, limited scalability, high cost, timeline constraints, reagent batch effects, contamination or stromal overgrowth, and the lack of standardized protocols and readouts. Biological incompleteness remains a fundamental limitation, as most models lack functional vasculature, fully integrated immune and neural compartments, systemic endocrine and pharmacokinetic regulation, and the ability to comprehensively capture spatial and temporal heterogeneity. At the translational level, unresolved issues include the need for prospective clinical validation, standardized definitions of actionable sensitivity, regulatory frameworks for living diagnostic assays, workflow integration into routine oncology practice, and demonstration of cost-effectiveness. Addressing these interrelated barriers is essential for advancing 3D cancer models from experimental platforms to robust, clinically deployable tools

Despite their transformative potential, the widespread adoption and clinical integration of 3D cancer models are impeded by significant and interrelated technical, biological, and translational hurdles. A clear-eyed assessment of these limitations is not a repudiation of the technology’s value but a necessary roadmap for guiding its future development, setting realistic expectations, and ensuring its rigorous application. The challenges span the entire workflow, from the foundational steps of model generation to the complexities of data interpretation and the final barrier of clinical validation (Fig. 4).

### Technical and logistical hurdles

The very strengths of 3D models such as their physiological complexity and fidelity introduce substantial technical barriers that affect reproducibility, scalability, and cost. The establishment of PDO biobanks remains a resource-intensive endeavor characterized by variable outcomes: success rates are not universal and can vary widely by tumor type and sample quality, with published examples ranging from about 60% in some prospective clinical implementations to above 80% in others [27, 236, 237]. The process demands specialized expertise and costly inputs, notably basement-membrane extracts such as Matrigel and recombinant growth factors, plus dedicated cell-culture infrastructure, all of which constrain scalability and make population-scale biobanking economically challenging. While tumor spheroid models are more accessible, generating large numbers of highly uniform spheroids for true high-throughput assays still often relies on specialized microplates, microwell or microarray-style platforms and associated automation or analysis workflows. Furthermore, the timeline for PDO establishment and drug testing can range from approximately 2 weeks in optimized workflows to about 6–8 weeks in many clinically oriented pipelines, which can conflict with urgent treatment decision windows in advanced cancers, though continued protocol and automation improvements are steadily shortening turnaround times [27, 132, 147, 238].

A major impediment to the field’s maturation is the pervasive lack of standardization. Media formulations, matrix types, and detailed protocols vary considerably between laboratories, leading to inter-study variability that complicates data comparison, meta-analysis, and the establishment of universal benchmarks. The well-documented batch-to-batch variability of key reagents like Matrigel introduces an uncontrolled confounder. There is a pressing need for the adoption of defined, synthetic matrices and serum-free media to improve reproducibility. Equally critical is the absence of standardized, validated readouts for key endpoints like viability, invasion, and drug response in 3D formats. Without consensus on how to measure success, it is difficult to compare results across platforms or establish clinically actionable thresholds for “sensitivity” or “resistance” [22, 239, 240].

One promising avenue toward standardization is the increasing availability of commercial, serum‑free media formulations specifically developed for PDO culture. Products such as IntestiCult (STEMCELL Technologies) for intestinal organoids, TumnoMACS (Miltenyi Biotec) for various tumor types, and OncoPro (available through multiple suppliers) provide defined, quality‑controlled components that reduce inter‑laboratory variability and improve reproducibility across different cancer types [62]. These commercial systems offer several advantages over custom‑formulated media: they eliminate batch‑to‑batch variability associated with in‑house reagent preparation, reduce the need for time‑consuming optimization of growth factor cocktails, and often include proprietary components that support long‑term maintenance of patient‑specific molecular characteristics. However, several limitations remain. First, these media are not universally applicable to all tumor types; certain cancers may require tumor‑specific modifications or completely different formulations. Second, the proprietary nature of these products means that the exact composition is not disclosed, which can hinder mechanistic studies aimed at understanding how specific medium components influence PDO biology. Third, the higher cost of commercial systems compared to custom media may limit their adoption in resource‑constrained settings. Despite these challenges, the growing adoption of commercial PDO culture systems represents an important step toward the standardization needed for clinical translation and multi‑center studies, and ongoing efforts to develop fully synthetic, defined matrices and media will further advance this goal.

Additional practical challenges include contamination and selective overgrowth. In PDO culture, a persistent issue is the overgrowth of normal epithelial organoids from adjacent healthy tissue within a sample, as well as stromal carryover or contamination such as fibroblasts that can confound downstream assays despite the use of selective conditions [241–243]. In advanced co-culture models designed to mimic the TME, maintaining the intended phenotypic states and stable proportions of multiple cell types, including immune cells, CAFs, cancer cells, over extended culture periods remains technically demanding because different compartments often require competing media requirements and exhibit differential survival or expansion kinetics [221, 225, 244].

### Biological complexity and incompleteness

Even the most advanced current 3D models are, by necessity, simplified representations of the immensely complex in vivo reality of human tumors. A primary limitation is the incomplete recapitulation of the TME: while co-culture and engineered systems have progressed rapidly, many widely used PDO formats still capture only a subset of the relevant non-malignant compartments [245, 246]. Key missing elements often include a functional, perfusable human vasculature, which is increasingly approached via vascularized organoid-on-chip and perfusion strategies but remains non-trivial to implement routinely at scale [247, 248]. A second major limitation is the absence of a fully integrated and spatially organized immune system. Although tumor organoid-immune co-culture platforms have enabled the incorporation of selected immune populations, such as tumor-infiltrating lymphocytes or engineered T cells, these systems typically lack the full diversity of myeloid, lymphatic, and bone marrow-derived compartments and do not recapitulate their native anatomical organization or systemic recruitment dynamics [201, 225]. In addition, the neuromodulatory component of tumor-innervating nerves is rarely modeled, even though tumor-nerve interactions are increasingly recognized as biologically relevant [249]. Finally, the lack of systemic circulation and endocrine signaling limits investigation of distal effects and true organ-level pharmacokinetics or pharmacodynamics, motivating integration with microphysiological and multi-organ-on-chip systems [250, 251]. Although organoid-based platforms are beginning to interrogate the microbiome-tumor-therapy axis, most cancer 3D models do not natively incorporate gut microbiome influences that can modulate treatment outcomes [252].

Another fundamental challenge lies in capturing dynamic spatial and temporal heterogeneity. A biopsy used to generate a PDO provides only a single snapshot of the tumor’s genetic and cellular landscape at one time point and from one location so it can miss geographically distinct clones within the primary tumor and, critically, subclones present at metastatic sites that may later dominate under therapy [239, 253]. While serial sampling and emerging liquid biopsy-based modeling approaches offer a potential avenue for longitudinally tracking tumor evolution with reduced invasiveness, their application remains technically challenging and not yet routine, owing to factors such as limited tumor cell yield, variable viability, and difficulties in establishing stable ex vivo cultures [37]. Therefore, modeling the full, multi-organ cascade of metastasis, namely from invasion and intravasation to survival in circulation, extravasation, and distant colonization, remains an immense challenge for current 3D platforms, although metastasis-on-a-chip or microphysiological systems are making meaningful inroads by enabling controlled flow, endothelial barriers, and organ-specific microenvironments within the same experimental framework [89, 254].

Finally, 3D in vitro models, by their nature, lack systemic physiology and therefore cannot replicate the integrated function of a whole organism. This limits their ability to model organ-level toxicities such as cardiotoxicity and hepatotoxicity, complex drug metabolism and clearance including first-pass hepatic effects, or systemic immune-microbiome-tumor interactions that shape outcomes in patients [181, 255]. Consequently, although 3D tumor models provide high-resolution insight into tumor-intrinsic and local microenvironmental responses, they remain fundamentally disconnected from organism-level pharmacology and physiology. Questions related to systemic toxicity, whole-body drug distribution, endocrine modulation, and microbiome-mediated immune effects therefore require complementary platforms that integrate multi-organ interactions beyond the scope of current tumor-restricted systems [250, 256, 257].

Given these substantial biological limitations, researchers might question how PDO-based drug screens can demonstrate clinical predictive value. The answer lies in recognizing that not all therapeutic questions require the same level of model complexity. For conventional cytotoxic chemotherapies and many targeted agents, the primary determinants of response, such as intrinsic proliferative capacity, apoptosis competence, and driver oncogene dependency, are largely cell-intrinsic and are preserved even in simplified epithelial-only organoid cultures. This explains why PDOs have shown strong predictive value for these drug classes, despite lacking immune and stromal components. Conversely, more complex TME features become critically important for other therapeutic contexts. For instance, immunotherapies including immune checkpoint inhibitors and CAR-T cells require co-culture systems that incorporate autologous or allogeneic immune effectors, as the tumor-immune interaction is the primary determinant of response. Moreover, stroma-targeting agents and drugs whose efficacy is modulated by matrix-mediated signaling such as integrin inhibitors and ECM-modifying enzymes necessitate co-culture with cancer-associated fibroblasts or engineered matrices that recapitulate desmoplastic features. In addition, resistance mechanisms involving stromal protection such as CAF-secreted survival factors or hypoxia-driven adaptations require engineered models that capture these microenvironmental contributions. Thus, a tiered approach to model selection is warranted: simpler PDO models suffice for predicting response to cell-autonomous drug classes, whereas progressively more complex systems including co-culture, organ-on-chip and bioprinted constructs are required for immunotherapies, stroma-modulating agents, and studies of microenvironment-mediated resistance.

### Translational and clinical barriers

Bridging the gap from a compelling research tool to a validated component of clinical practice presents a distinct set of obstacles rooted in evidence generation, regulation, and healthcare logistics [155]. For PDOs to be adopted as clinical decision-support tools, they must demonstrate clinical utility through prospective interventional trials, and ultimately randomized comparisons of PDO-guided treatment versus standard-of-care pathways with clinically meaningful endpoints such as progression-free survival (PFS) or overall survival (OS) [258, 259]. While early retrospective work and small prospective feasibility or intervention studies support plausibility and operational feasibility, definitive, adequately powered trials are expensive and logistically complex, and many are still ongoing or only recently registered [260, 261]. Concurrently, critical operational questions remain: what constitutes a clinically actionable “positive” result, and what thresholds define sensitivity or resistance, and how should failed or inconclusive cultures be handled within a real-world clinical algorithm [262, 263].

The regulatory pathway for complex living models like PDOs is less established than for traditional molecular diagnostics, but important parallels can be drawn with antimicrobial susceptibility testing (AST). In AST, live bacterial cultures isolated from patient specimens are routinely tested against panels of antibiotics to guide therapy, a paradigm that shares core features with PDO-based drug testing, including the need for robust quality control, standardized reference strains, interpretative criteria for “sensitive” versus “resistant” classifications, and rapid turnaround times. The regulatory frameworks developed for AST over decades, including CLSI guidelines and FDA clearance pathways for susceptibility testing devices, provide a potential roadmap for PDO-based assays [264, 265]. Recent FDA recognition of CLSI breakpoints for hundreds of organism-antimicrobial combinations demonstrates the feasibility of aligning consensus standards with regulatory requirements [266].

That said, regulatory agencies have extensive experience with genetic and protein-based companion diagnostics, but a framework for clinical-grade PDO generation, which ensures analytical validity, clinical validity, and clinical utility, must still be developed. This involves creating standards for every step, from sample acquisition and transport under chain-of-custody to cell culture, drug testing, and final reporting, all under a quality management system akin to CLIA certification for laboratory tests. Developing such standards for emerging in vitro test methods, including organoid-based assays, is an acknowledged regulatory challenge because existing frameworks were not designed with living diagnostic cultures in mind [267]. Collaboration between academia, industry, and regulatory bodies is essential to build this novel framework [155]; while leveraging lessons from both molecular diagnostics and AST will be critical to this effort.

Finally, for functional precision medicine to become routine, it must integrate seamlessly into existing clinical workflows and prove its cost-effectiveness. This requires solving logistical challenges such as establishing reliable networks for rapid sample processing and transport from community hospitals to specialized laboratories, guaranteeing fast and reliable turnaround times for results, and creating clear channels for communicating complex functional data to treating oncologists. A robust health-economic analysis is ultimately required to prove that the significant upfront cost of PDO testing is offset by downstream savings from avoiding ineffective, toxic treatments and from improving patient outcomes and survival. Demonstrating this value proposition is critical for securing reimbursement from healthcare payers and enabling widespread access.

## Perspectives and conclusions

**Fig. 5:**
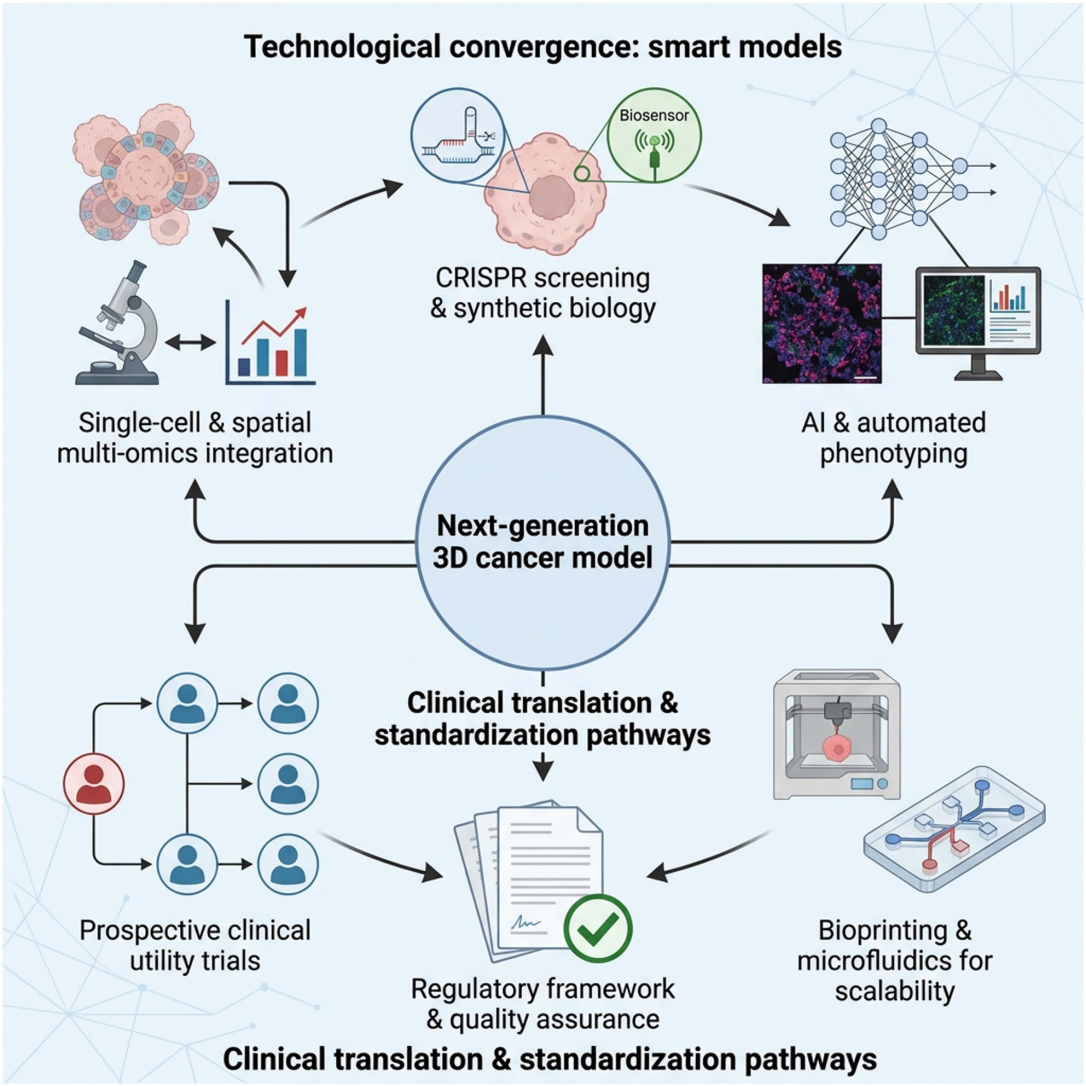
Technological convergence toward next-generation 3D cancer models. Emerging framework for the development of “smart” 3D cancer systems through integration of advanced biological engineering, multi-omic analytics and clinical standardization. Coupling patient-derived and engineered tumor models with single-cell and spatial multi-omics enables high-resolution mapping of cellular ecosystems and treatment-induced state transitions. Integration with CRISPR-based functional genomics, synthetic biology and embedded biosensors facilitates systematic perturbation and real-time pathway monitoring within physiologically relevant 3D contexts. Artificial intelligence-driven image analysis and automated phenotyping transform multidimensional morphological and molecular outputs into predictive signatures of drug response. In parallel, advances in bioprinting and microfluidic platforms aim to enhance scalability and reproducibility, supporting standardized workflows. Translation into routine oncology practice requires prospective clinical utility trials, regulatory frameworks and quality assurance systems to establish analytical validity, clinical validity and cost-effectiveness. Together, these converging technological and translational pathways define the roadmap for next-generation 3D cancer models with integrated predictive and clinical capabilities

The trajectory of 3D cancer modeling is unequivocally pointing toward systems of greater biological fidelity, deeper analytical power, and tighter clinical integration. The future of this field lies not merely in the incremental improvement of isolated platforms but in the strategic convergence of diverse technologies and the systematic, evidence-based translation of rigorously validated models into robust clinical tools. We here outline the most promising horizons for the next generation of models and reiterates the transformative potential of this paradigm in redefining both cancer research and patient care (Fig. 5).

From a technological perspective, one of the foundational trends is the integration with single-cell and spatial multi-omics. The future standard for model characterization will move beyond bulk molecular confirmation to routine single-cell or spatial transcriptomic, proteomic, and epigenomic profiling. This will enable a nuanced understanding of how a model recapitulates the cellular ecosystem and spatial organization of a tumor. For instance, performing single-cell RNA sequencing on PDOs before, during, and after drug exposure will allow researchers to track the fate of specific subclones with unprecedented precision, mapping trajectories of cell state transitions toward resistance or death. Similarly, coupling 3D models with CRISPR screening and synthetic biology will unlock systematic discovery. Performing genome-wide CRISPR knockout or activation screens within a diverse PDO biobank will map genetic interactions and vulnerabilities across tumor genotypes in a physiologically relevant 3D context, revealing targets that would be missed in 2D. Furthermore, embedding synthetic biosensors into organoids to report on specific pathway activities or cell-cell communication signals in real-time will enable dynamic, non-destructive monitoring of biological processes. Another impactful integration will be with artificial intelligence and automated phenotyping. The complexity of data generated by advanced 3D models exceeds the analytical capacity of traditional methods. Deep learning algorithms can be trained to analyze microscopy images, quantifying subtle morphological changes, classifying organoid subtypes, and even predicting drug response based on phenotypic features alone. This moves the analytical paradigm from simple endpoint viability measurements to rich, multidimensional phenotypic profiling, enabling the discovery of novel structure-activity relationships and the identification of predictive morphological signatures that precede cell death. Together, the next qualitative leap in 3D cancer modeling will emerge from the deep integration of these biological platforms with cutting-edge analytical and manipulative tools, thereby creating “smart” systems that are both highly representative and profoundly interrogable.

For the ambitious promise of functional precision medicine to be realized, 3D models must successfully navigate the path from research to regulated clinical application, a journey requiring both scientific validation and operational innovation. The most critical immediate step is the transition from feasibility studies to demonstrations of clinical utility in prospective trials. Large-scale, randomized controlled trials are essential to definitively answer whether therapy selection guided by PDO testing leads to superior patient outcomes such as improved progression-free or overall survival compared to standard-of-care selection such as genomics-guided or empiric therapy. Success in such trials is the non-negotiable prerequisite for widespread clinical adoption and would represent a watershed moment for the field. In parallel, a regulatory and quality assurance framework must be co-developed. Regulatory agencies, researchers, and diagnostic companies need to collaborate to establish a pathway for PDO-based tests. This involves developing consensus standards for clinical-grade model generation including tissue acquisition, processing, culture, and drug testing, defining clear analytical and clinical performance metrics including sensitivity, specificity, predictive values, and implementing rigorous quality management systems to ensure reproducibility across different clinical laboratories. To address the scalability challenge, future platforms may leverage bioprinting and microfluidics for scalable personalized testing. Robotic bioprinting could rapidly array hundreds of standardized, miniature tumor constructs from a patient’s cells for simultaneous testing of dozens of drug conditions. Similarly, integrated microfluidic “lab-on-a-chip” devices could automate the entire process from sample-in to result-out within a single chip, drastically reducing hands-on time, cost, and turnaround time, thereby making personalized tumor avatars accessible to a broader patient population.

In summary, the advent and refinement of sophisticated 3D cancer models, spearheaded by PDOs, unequivocally marks a paradigm shift in oncology. These systems directly address the core deficiency of previous models by preserving patient-specific tumor architecture, genetics, and, most importantly, functional response profiles within a controllable in vitro environment. As detailed in this review, they have proven their worth as unparalleled tools for deconstructing the complex biology of therapy resistance, for empirically guiding personalized treatment selection, and for accelerating and de-risking the drug discovery pipeline by providing human-relevant efficacy and toxicity data much earlier in the development process. The journey from a self-organizing cellular aggregate to a predictive clinical tool is undeniably complex, fraught with the technical, biological, and translational challenges outlined herein. However, the direction of travel is clear. Through continued technological innovation, unwavering commitment to standardization and reproducibility, and rigorous clinical validation, both generic 3D cancer models and PDOs are poised to fulfill their distinct but complementary potentials. Generic 3D models, particularly tumor spheroids, excel in high-throughput mechanistic studies, first-pass drug screening, and assays of drug penetration and hypoxia-driven resistance, owing to their scalability and compatibility with automated workflows. PDOs, by contrast, offer unparalleled patient-specific fidelity, preserving the genetic, molecular, and histopathological features of the source tumor, making them indispensable for personalized medicine, subtype-specific biology, and modeling acquired resistance. Recognizing these distinct roles will be critical for aligning model selection with specific research and translational questions. PDOs, in particular, stand to transform oncology from a discipline that often relies on population-level probabilities and static genomic snapshots to one increasingly capable of delivering dynamic, patient-specific therapeutic insights based on the living biology of a tumor. In doing so, they offer a tangible and powerful path toward the long-sought goal of truly personalized cancer medicine, where the optimal treatment strategy for an individual is determined not only by the sequence of their tumor’s DNA but by the observable response of their own living tumor avatar in the laboratory.

## Data Availability

No datasets were generated or analysed during the current study.
